# Lipid phenotyping of lung epithelial lining fluid in healthy human volunteers

**DOI:** 10.1007/s11306-018-1412-2

**Published:** 2018-09-17

**Authors:** Joost Brandsma, Victoria M. Goss, Xian Yang, Per S. Bakke, Massimo Caruso, Pascal Chanez, Sven-Erik Dahlén, Stephen J. Fowler, Ildiko Horvath, Norbert Krug, Paolo Montuschi, Marek Sanak, Thomas Sandström, Dominick E. Shaw, Kian Fan Chung, Florian Singer, Louise J. Fleming, Ana R. Sousa, Ioannis Pandis, Aruna T. Bansal, Peter J. Sterk, Ratko Djukanović, Anthony D. Postle, The U-BIOPRED Study Group, I. M. Adcock, H. Ahmed, C. Auffray, P. Bakke, A. T. Bansal, F. Baribaud, S. Bates, E. H. Bel, J. Bigler, H. Bisgaard, M. J. Boedigheimer, K. Bønnelykke, J. Brandsma, P. Brinkman, E. Bucchioni, D. Burg, A. Bush, M. Caruso, A. Chaiboonchoe, P. Chanez, F. K. Chung, C. H. Compton, J. Corfield, A. D’Amico, B. Dahlén, S. E. Dahlén, B. De Meulder, R. Djukanović, V. J. Erpenbeck, D. Erzen, K. Fichtner, N. Fitch, L. J. Fleming, E. Formaggio, S. J. Fowler, U. Frey, M. Gahlemann, T. Geiser, V. Goss, Y. Guo, S. Hashimoto, J. Haughney, G. Hedlin, P. W. Hekking, T. Higenbottam, J. M. Hohlfeld, C. Holweg, I. Horváth, P. Howarth, A. J. James, R. G. Knowles, A. J. Knox, N. Krug, D. Lefaudeux, M. J. Loza, R. Lutter, A. Manta, S. Masefield, J. G. Matthews, A. Mazein, A. Meiser, R. J. M. Middelveld, M. Miralpeix, P. Montuschi, N. Mores, C. S. Murray, J. Musial, D. Myles, L. Pahus, I. Pandis, S. Pavlidis, A. Postle, P. Powel, G. Praticò, M. Puig Valls, N. Rao, J. Riley, A. Roberts, G. Roberts, A. Rowe, T. Sandström, J. P. R. Schofield, W. Seibold, A. Selby, D. E. Shaw, R. Sigmund, F. Singer, P. J. Skipp, A. R. Sousa, P. J. Sterk, K. Sun, B. Thornton, W. M. van Aalderen, M. van Geest, J. Vestbo, N. H. Vissing, A. H. Wagener, S. S. Wagers, Z. Weiszhart, C. E. Wheelock, S. J. Wilson

**Affiliations:** 1 Clinical and Experimental Sciences, Faculty of Medicine, University of Southampton, Southampton, UK; 2 Data Science Institute, Imperial College, London, UK; 3 Department of Clinical Science, University of Bergen, Bergen, Norway; 4 Department of Clinical and Experimental Medicine, University of Catania, Catania, Italy; 5 Department of Respiratory Diseases, Aix-Marseille University, Marseille, France; 6 Institute of Environmental Medicine, Karolinska Institute, Stockholm, Sweden; 7 Division of Infection, Immunity and Respiratory Medicine, School of Biological Sciences, The University of Manchester, Manchester, UK; 8 Manchester Academic Health Science Centre, University Hospital of South Manchester, Manchester, UK; 9 Department of Pulmonology, Semmelweis University, Budapest, Hungary; 10 Fraunhofer Institute for Toxicology and Experimental Medicine, Hannover, Germany; 11 Department of Pharmacology, Faculty of Medicine, Catholic University of the Sacred Heart, Rome, Italy; 12 Department of Medicine, Jagiellonian University, Krakow, Poland; 13 Department of Public Health and Clinical Medicine, Umeå University, Umeå, Sweden; 14 Respiratory Research Unit, University of Nottingham, Nottingham, UK; 15 National Heart and Lung Institute, Imperial College, London, UK; 16 University Children’s Hospital Bern, Bern, Switzerland; 17 Respiratory Therapy Unit, GlaxoSmithKline, London, UK; 18 Acclarogen Ltd, St John’s Innovation Centre, Cambridge, UK; 19 Academic Medical Center, University of Amsterdam, Amsterdam, The Netherlands; 20 National Institute for Health Research Southampton Biomedical Research Centre, Southampton, UK

**Keywords:** Induced sputum, Epithelial lining fluid, Pulmonary surfactant, Lipid metabolism, Lipidomics, Mass spectrometry, Weight status

## Abstract

**Background:**

Lung epithelial lining fluid (ELF)—sampled through sputum induction—is a medium rich in cells, proteins and lipids. However, despite its key role in maintaining lung function, homeostasis and defences, the composition and biology of ELF, especially in respect of lipids, remain incompletely understood.

**Objectives:**

To characterise the induced sputum lipidome of healthy adult individuals, and to examine associations between different ELF lipid phenotypes and the demographic characteristics within the study cohort.

**Methods:**

Induced sputum samples were obtained from 41 healthy non-smoking adults, and their lipid compositions analysed using a combination of untargeted shotgun and liquid chromatography mass spectrometry methods. Topological data analysis (TDA) was used to group subjects with comparable sputum lipidomes in order to identify distinct ELF phenotypes.

**Results:**

The induced sputum lipidome was diverse, comprising a range of different molecular classes, including at least 75 glycerophospholipids, 13 sphingolipids, 5 sterol lipids and 12 neutral glycerolipids. TDA identified two distinct phenotypes differentiated by a higher total lipid content and specific enrichments of diacyl-glycerophosphocholines, -inositols and -glycerols in one group, with enrichments of sterols, glycolipids and sphingolipids in the other. Subjects presenting the lipid-rich ELF phenotype also had significantly higher BMI, but did not differ in respect of other demographic characteristics such as age or gender.

**Conclusions:**

We provide the first evidence that the ELF lipidome varies significantly between healthy individuals and propose that such differences are related to weight status, highlighting the potential impact of (over)nutrition on lung lipid metabolism.

**Electronic supplementary material:**

The online version of this article (10.1007/s11306-018-1412-2) contains supplementary material, which is available to authorized users.

## Introduction

**Fig. 1:**
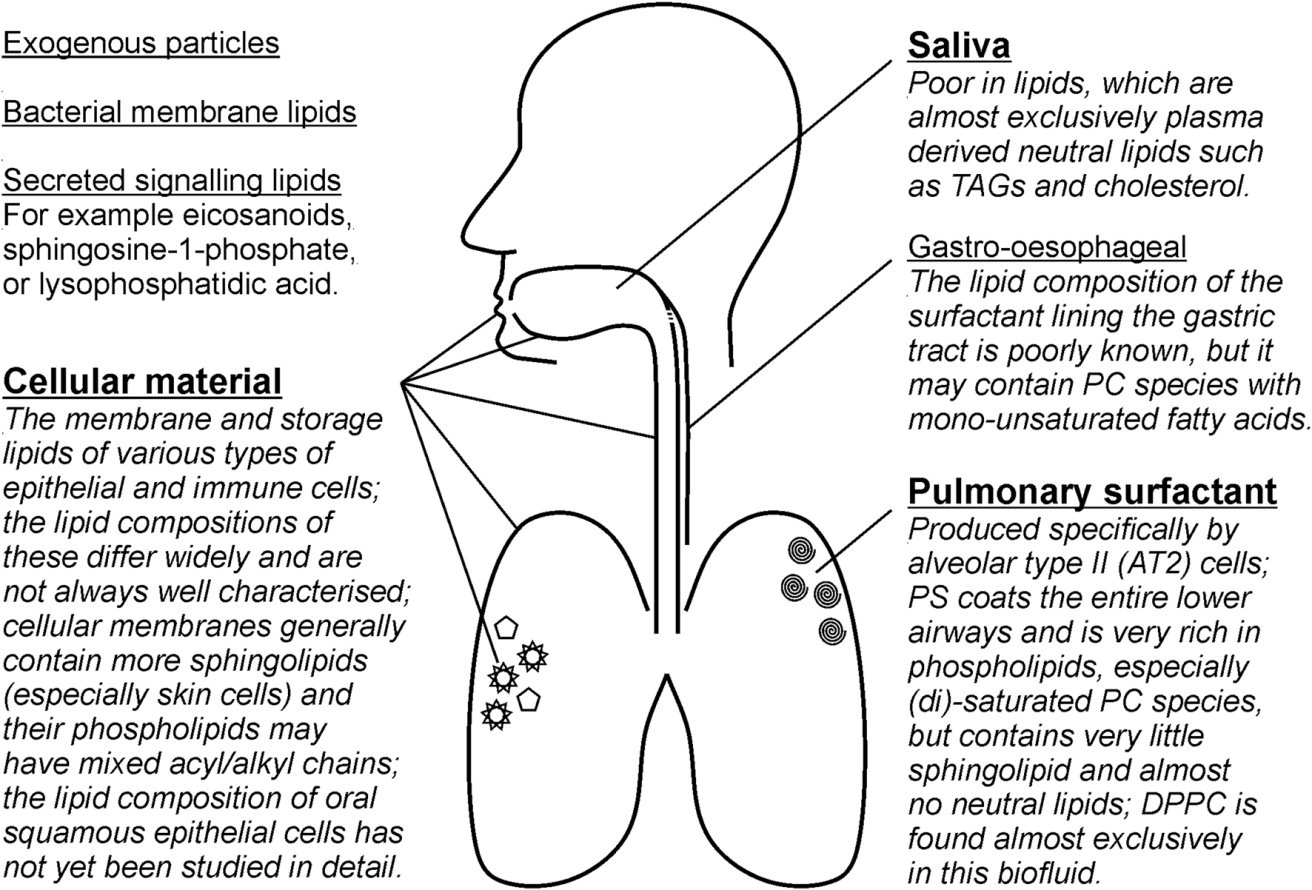
Induced sputum is a composite sample that, whilst the procedure targets the lower airways, contains material from a range of sources. The relative contributions of each of these vary and this complicates the robust measurement of biomarkers in induced sputum samples. This figure summarises the main sources of material in induced sputum (in bold), as well as some potential minor sources, and gives some of the characteristics of the lipids found in each source (for more detail see Kremlev et al. 1994; Larsson et al. 1996; Fessler and Summer 2016)

Epithelial lining fluid (ELF) is the thin layer of biofluid that covers the apical surface of the respiratory epithelium, from the alveoli up through to the large airways. As the first barrier between the lung and the external environment, it is a prime target for molecular studies of lung disease. Sputum induction is a non-invasive procedure for sampling ELF (Chanez et al. 2002) and is widely used to study the pathobiological mechanisms, inflammatory responses and microbial compositions of respiratory diseases such as asthma (Seys 2017; Wright et al. 2000), chronic obstructive pulmonary disease (COPD) (Iwamoto et al. 2014; Shaw et al. 2014; Telenga et al. 2014), cystic fibrosis (Muhlebach and Sha 2015; Quinn et al. 2016; Sagel et al. 2007) and tuberculosis (Pan et al. 2015). Although sputum induction primarily targets secretions which originate in the lower airways, sputum samples actually comprise a mixture of pulmonary surfactant, saliva, immune cells, and squamous cells from the upper airways and oral epithelium (Fig. 1). The ratio of each of these varies depending on the subject, disease state and the induction method used (e.g. Belda et al. 2000; Pizzichini et al. 1996). Moreover, sputum samples may contain secretions from the upper airways or the gastroesophageal tract, inhaled aerosols and microbes. These additional sources can affect the concentrations of lower airways biomarkers, principally through dilution of the sample with saliva, and may also influence its molecular composition.

Despite the widespread use of induced sputum in lung research, its lipid composition and the associated influencing biochemical factors have not been fully elucidated. In part, this may be due to the difficulty in obtaining sufficient good quality samples, particularly from healthy individuals (Chanez et al. 2002; Lins 2016). The few studies that have described the lipid composition of ELF (as sampled by sputum induction) have shown that it is dominated by glycerophospholipids, in particular a restricted number of saturated glycerophosphocholine (PC) species, with only small amounts of other classes such as glycerophosphoglycerol (PG), -ethanolamine (PE) and -inositol (PI) being present (Dushianthan et al. 2012; Sahu and Lynn 1978; t’Kindt et al. 2015). A variety of glycerolipids (di- and triacylglycerols together with free fatty acids), sterols (predominately cholesterol) and sphingolipid species (various species of ceramides and sphingomyelins) have also been detected, but these are much less abundant in induced sputum than in plasma or tissue samples (Sahu and Lynn 1978; t’Kindt et al. 2015). While these existing studies have offered valuable insight into the molecular diversity of induced sputum samples, more information on the amounts and variability of individual lipid molecular species is required. The lipid composition of any biofluid can be significantly affected by inherent instrumental and biological variabilities between samples and individuals (e.g. Sales et al. 2016). Indeed, the relative abundances of PC species have been shown to differ between induced sputum, BAL and tracheal wash samples, as well as between patients (Dushianthan et al. 2012). Such variability must be considered in respect of sputum, particularly when this sampling method is used for large cohort-based disease studies (Hyötyläinen and Orešič 2015).

In view of the limited knowledge about the pulmonary lipidome under ‘normal’ conditions, the aims of the current study were to: (1) define better the lipid composition of induced sputum (and by extension ELF), in a substantial cohort of healthy non-smoking adult volunteers; (2) determine the variability of the ELF lipidome within this population; and (3) examine whether any observed lipidomics differences are associated with common demographic and physiological parameters.

## Materials and methods

A more extensive description of the study design, sample collection, experimental procedures and data analysis methods is presented in Supplementary Material 1.

### Study participants

All induced sputum samples used in this study were obtained from the U-BIOPRED cohort (*Unbiased Biomarkers for the Prediction of Respiratory Disease Outcomes*). A total of 101 healthy non-smoking individuals were recruited in U-BIOPRED, of whom 55 successfully provided a sputum sample from either of two inductions. Of these, 41 passed the QC criteria for analysis based on cell viability, resuspension volume and a squamous epithelial cell cut-off of ≤ 40% (Shaw et al. 2015), and were included here. The study group comprised 29 males and 12 females of predominately Caucasian origin and recruited at different clinical centres across Europe; their demographic characteristics are summarised in Table 1.

**Table 1:** Characteristics of the healthy, non-smoking adults who provided induced sputum samples for this study

	Study group (*n* = 41)	TDA group 1 (*n* = 23)	TDA group 2 (*n* = 18)
Gender (male/female)	29/12 [71/29]	17/6 [74/26]	12/6 [67/33]
Ethnicity (Caucasian/Non-Caucasian)	37/4 [90/10]	22/1 [96/4]	15/3 [83/17]
Age (years)	33 [18–65]	32 [23–65]	36 [18–50]
Height (cm)	177 [151–196]	176 [151–193]	177 [158–196]
Weight (kg)	80.0 [48.1–111.9]	75.2 [48.1–107.0]	83.5 [60.6–111.9]
Body mass index (kg/m^2^)	25.6 [18.9–32.0]	23.5 [18.9–32.0]	26.7 [22.8–30.8]
FEV1 (% predicted)	102.6 [66.9–123.6]	98.8 [66.9–123.6]	109.8 [79.6–122.9]
Atopy (positive/negative/unknown)	14/18/9 [34/44/22]	9/11/3 [39/48/13]	5/7/6 [28/39/33]

The values for gender, ethnicity and atopy status are shown as counts and percentages, whereas the results for age, weight, height, BMI and FEV1 are given as median values and ranges

### Lipid extraction

Lipids were extracted from 100 µl of sputum using a semi-automated Bligh–Dyer protocol (Bligh and Dyer 1959) on a robotic liquid handling platform. Briefly, each sample was extracted using 700 µl of 0.9% saline solution, 2 ml of methanol (MeOH) and 1 ml of dichloromethane (DCM). Anti-oxidant (10 µl of 5 mg ml^−1^ butylated hydroxytoluene in MeOH) and synthetic lipid standards for internal quantification were added, followed by centrifugation at 3000 rpm to remove precipitated protein. Additional DCM and ultrapure water (1 ml each) were then added to the supernatants, followed by a second centrifugation (3000 rpm) step. The lower organic phase was recovered, dried under a stream of N_2_ gas and stored at − 80 °C until analysis.

### Lipid analysis

Samples were reconstituted in 1 ml of MeOH:DCM:50 mM aqueous NH_4_HCO_2_ (50:50:8 v/v), and 20-µl aliquots were removed from each sample and pooled to create a quality control (QC) sample. All measurements were performed on a MaXis 3G high resolution quadrupole time-of-flight mass spectrometer equipped with an electrospray ionization source (Bruker Daltonics, Bremen, Germany), coupled to an UltiMate 3000 ultra-high performance liquid chromatography system (Dionex, Sunnyvale, CA, USA). For the initial screening by untargeted ‘shotgun’ MS, samples were introduced by loop injection into a continuous stream of MeOH and mass spectra were acquired in full scan mode over an *m*/z range of 350–1200 (with separate injections for positive and negative ionisation). Blank injections were performed after every four samples (no significant carry-over was detected) and the pooled QC sample was run after every four samples to check for changes in instrument performance.

Fragmentation analysis for lipid identification was performed using the same instrumental setup but in LC–MS/MS mode. Samples (10 µl injection) were first separated on a C8 column (Waters Acquity UPLC CSH C8, 130 Å, 1.7 μm, 2.1 mm × 100 mm) using mobile phases of (A) MeOH with 50 mM NH_4_HCO_2_ and 0.2% formic acid, and (B) 50 mM aqueous NH_4_HCO_2_ with 0.2% formic acid (all LC grade). The following gradient was used: linear increase from 80 to 98% A at 0.3 ml min^−1^ over the first 10 min, then a linear increase to 100% A at 0.3 ml min^−1^ over the next 10 min, isocratic at 100% A for 25 min but with an increased flow rate of 0.4 ml min^−1^, rapid return to the starting conditions (80% A at 0.3 ml min^−1^) to re-equilibrate the system for 5 min. Data-independent product ion scans were acquired over the entire 50 min gradient using the bbCID (broadband Collision Induced Dissociation) function in Compass (Bruker Daltonics). In this setting, the MS rapidly alternates between low and high collision energy, resulting in parallel sets of intact precursor and fragment ions over the selected mass range. This allows for precursor and fragment ions to be matched retrospectively by their LC retention times and peak elution patterns and using well-established fragmentation rules for lipids (Hsu and Turk 2003) to provide confirmation of identities.

### Data processing

All screening mass spectra were smoothed and lock mass calibrated using the internal standard peaks. An averaged “background” spectrum and “sample” spectrum were generated from each sample injection and exported as separate mass-intensity lists. The ions in all mass-intensity lists were aligned using a hierarchical clustering-based algorithm (adapted from Yang 2016); detailed description in Supplementary Material 1). After alignment, individual “background” spectra were subtracted from their associated “sample” spectra and, additionally, an average spectrum from all of the blank runs was also subtracted from each of the “sample” spectra. This protocol excluded from the final results all possible background signals from the instrument or introduced during sample preparation and storage. For each sample the average number of counts of each ion was calculated based on triplicate injections, with ions present in only one out of three runs removed from the list. Moreover, ions with < 60% detection rate across all samples were excluded from further analysis. To control for the introduction of batch effects due to instrument performance or differences in sample work-up date all results were run through the widely used R script “ComBat” (Johnson et al. 2007).

All ion counts were normalised to the signal intensity of the 1,2-dimyristoyl-*sn*-glycero-3-phosphocholine (DMPC) internal standard, as well as the original sample volume, to obtain semi-quantitative results (i.e. µM relative to DMPC). However, biofluids and particularly induced sputum are subject to variable dilution of analytes during sampling and subsequent workup (Simpson et al. 2004). As such variations can mask interesting trends or patterns within the dataset, a normalisation step is often required to compensate for sample dilution (Kirwan et al. 2014). Normalisation of MS-based data is generally done as a fraction of the total signal, or relative to a specific ‘housekeeping’ protein or metabolite. We opted for the latter and used 1,2-dipalmitoyl-*sn*-glycero-3-phosphocholine (DPPC) as a biomarker for lower airways secretions and, by extension, sample dilution. The rationale is that DPPC is produced in large quantities by the alveolar type II cells and is the most abundant lipid in pulmonary surfactant (Brandsma and Postle 2017; Goss et al. 2012), whereas it is not a major component of the blood-derived salivary lipidome (Larsson et al. 1996) or of cellular membranes.

### Statistical analysis

Topological data analysis (TDA) was used to visualise groups of participants within the study cohort with comparable sputum lipid profiles in an unbiased manner. TDA was performed using the Ayasdi machine intelligence platform (Ayasdi, Palo Alto, CA, USA) on the selected lipid data set (291 ions) and employing a normalised correlation metric combined with multidimensional scaling (MDS) lenses. Discreet groups of participants were defined manually within the TDA networks as previously reported (Bigler et al. 2016; Hinks et al. 2016). Lipid composition and participant metadata (such as age, gender, BMI, cell counts) between each of the selected subgroups were then compared by Mann–Whitney U test with a significance threshold of *p* < .05. No adjustments for multiple testing were done because of the relatively small cohort size, and validation of the results in follow-up studies is therefore warranted.

## Results

### Sputum samples and cell counts

All 41 sputum samples were obtained from healthy non-smoking participants in the U-BIOPRED study and had passed the QC criteria for analysis listed above. The differential cell counts (Table 2) were dominated by macrophages and to a lesser degree neutrophils, with only small numbers of lymphocytes and almost no eosinophils, comparable to previous findings in cohorts of healthy non-smoking adults (Belda et al. 2000; Spanevello et al. 2000).

**Table 2:** Differential cell counts of the sputum samples given as mean values and ranges; note that the squamous epithelial cells are measured relative to the sum of the other cell types

	Study group (*n* = 41)	TDA group 1 (*n* = 23)	TDA group 2 (*n* = 18)
Macrophages	60.3 [8.0–96.3]	61.9 [8.0–96.3]	58.5 [25.8–93.9]
Neutrophils	38.0 [2.7–89.9]	36.4 [2.7–89.9]	39.7 [6.0–73.1]
Lymphocytes	1.2 [0.2–7.8]	0.9 [0.2–3.6]	1.4 [0.2–7.8]
Eosinophils	0 [0–1.6]	0.2 [0–1.6]	0 [0–1.1]
Squamous epithelial cells	12.7 [0–39.2]	18.1 [0.8–39.2]	5.8 [0–38.1]

### Lipid identification

A total of 1364 positive and 1031 negative ions were detected in the 41 analysed sputum samples, the majority being present in a small number of samples but absent or below the limit of detection in the remainder. Only the 291 ions detected in 60% or more of any of the samples were used for statistical analysis (see Supplementary Material 1 for additional discussion). Identities of more than half of these ions (which constituted 95% of the total signal) were confidently assigned through a combination of accurate mass, MS/MS fragmentation, LC retention time, and comparison with the Lipid MAPS online database and the list of sputum lipids presented in t’Kindt et al. (2015). For a complete overview of detected ions, their assignments and abundances, see the Table in Supplementary Material 2.

The most common lipid class both in terms of the number of assigned molecular species and concentration was PC (Table 3). A total of 7 lyso-PCs, 28 diacyl-PCs and 15 mixed alkyl/acyl-PCs were identified, comprising on average around 70% of the total signal. DPPC made up 33% of the total lipid, whilst other relatively abundant species all also contained palmitic acid: 3.9% PC[16:0/14:0] at *m*/*z* 706.539, 3.8% PC[16:0/16:1] at *m*/*z* 732.554, 2.2% PC[16:0/18:2] at *m*/*z* 758.568 and 6.0% PC[16:0/18:1] at *m*/*z* 760.585 (all detected in positive ESI). The second most common lipid class was PE, with 22 identified molecular species, but none in relative abundances exceeding more than 1% of total lipid. PG, PI and glycerophosphoserine (PS) were present in low concentrations, and each was represented by a few molecular species containing combinations of palmitic, palmitoleic, oleic and stearic acid. Only one diacylglycerol was found (DG[34:1] at *m*/*z* 612.575), but the sputum samples contained at least 11 different TG species, ranging from saturated TG[50:0] at *m*/*z* 852.787 to arachidonic acid-containing TG[54:4] at *m*/*z* 900.792. Concentrations of all glycerolipids were low. Cholesterol was present in relatively small amounts (< 5% of total lipid), as were three cholesteryl esters (CE). Finally, the samples contained a variety of sphingolipids: four d18:1-ceramides (Cer), two hexosyl-d18:1-ceramides (HexCer), and six sphingomyelins. As with the glycerophospholipids, the sphingolipids contained a variety of saturated and mono-unsaturated fatty acids, and they were generally present in small amounts (up to 1.9% of total lipid in the case of SM[d18:1/16:0] at *m*/*z* 703.572).

**Table 3:** Lipid classes, number of species and relative abundances detected in induced sputum samples

Lipid class	Identified lipid species per class	Average abundance of lipid class (%)
Glycerophosphocholines (PC)	50	71.2
Glycerophosphoethanolamines (PE)	22	9.8
Glycerophosphoglycerols (PG)	3	8.0
Glycerophosphoserines (PS)	1	1.9
Glycerophosphoinositols (PI)	2	0.1
Diacylglycerols (DG)	1	0.1
Triacylglycerols (TG)	11	0.8
Sterol lipids (ST)	5	1.4
Ceramides (Cer)	5	0.3
Glucosylceramides (GlcCer)	2	0.4
Sphingomyelins (SM)	6	0.9
Unidentified lipids	94	5.2

A total of 291 ions were detected in 60% or more of the sputum samples. Over half of these could be confidently assigned to a specific lipid class. However, the majority of unidentified ions were of low intensity, and in concentration terms PC, PE and PG species constituted almost 90% of the total lipid signal

### Topological data analysis

**Fig. 2:**
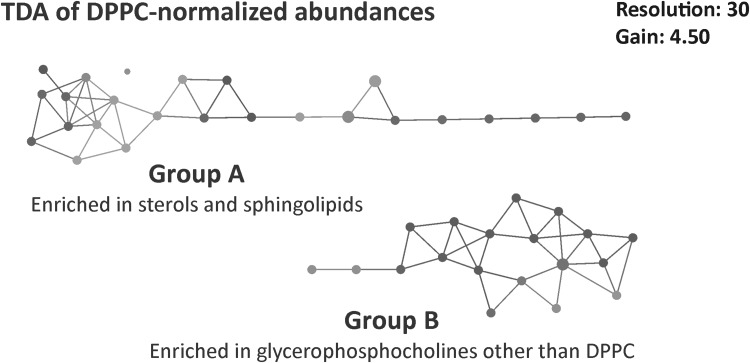
TDA networks of DPPC-normalised lipid abundances (bottom) show a consistent presence of two groups within the healthy non-smoking sputum sample set. TDA was performed on 291 ions and use a normalised correlation metric and two MDS lenses. The network is coloured by the total amount of lipid measured (average value of the samples in each node), with blue indicating low, and red high concentrations. The figure was obtained with the Ayasdi machine intelligence platform (http://www.ayasdi.com/platform)

**Fig. 3:**
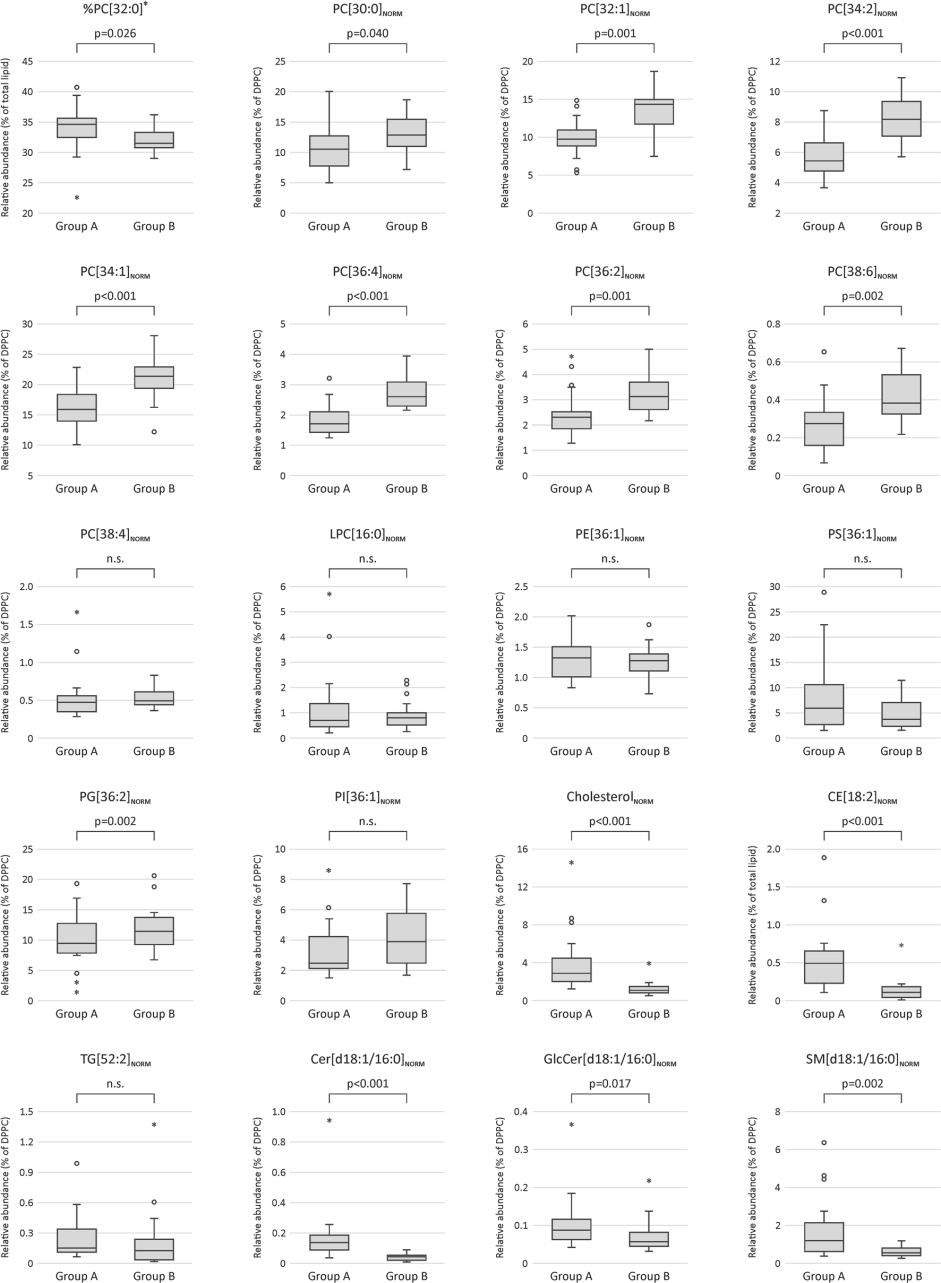
Comparison of the 291 lipid species between the two TDA groups showed significant differences, a selection of which are presented here (see Table in Supplementary Material 2 for a full comparison of all ions). Each box contains a comparison between groups A and B as identified by topological data analyses of the DPPC-normalised data. The exception is the relative abundance of DPPC itself (‡), for which the concentration was normalised to that of the total lipid signal. Boxplots were created in SPSS Statistics 24 (IBM) which defines outliers as ‘near’ (open circles: more than 1.5 times the interquartile range) and ‘far’ (stars: more than 3 times the interquartile range)

TDA of the DPPC-normalised lipid data yielded two distinct groups of study participants (Fig. 2). The larger of the two, group A (*n* = 25), was significantly enriched in cholesterol, CE species and sphingolipids (Cer and SM), as well as a small number of low-abundance glycerophospholipids (Fig. 3 and Supplementary Material 2). In contrast, group B (*n* = 16) was significantly enriched in all the major diacyl-PC species, but not the lyso-PCs or mixed acyl/alkyl-PCs, which were comparable between the groups. Interestingly, the relative abundance of DPPC itself (measured as % of total lipid) was actually lower in group B than in group A. The results for the PE class were mixed, with a few species being enriched in either group, but the majority did not differ. Group A was somewhat enriched in PS (not significant), whereas group B had higher PI and PG levels, although this was only significant for one of the three identified PG species. A second TDA was done on the non-normalised lipid data, which again yielded two distinct groups (Supplementary Material 1, Fig. S1). This distribution of samples had 90% similarity with the TDA groups of the DPPC-normalised data. Differential feature analysis showed that the grouping was driven by differences in overall lipid concentrations between A and B, which were significant for 71% of the ions (Supplementary Material 2). The main exception were the TG, CE and Cer species, almost all of which had comparable concentrations across all the sputum samples.

Comparison of the demographic and physiological characteristics of the study participants showed a significantly (*p* = .010) higher BMI in group B (median 26.7; range 23–31 kg m^−2^) than in group A (median 23.5; range 19–32 kg m^−2^), but no differences in respect of gender, ethnicity, age, weight, height lung function or atopic status (Table 1). To examine the association with BMI, the significance of the lipid differences between subjects with BMI > 25 (*n* = 22) and those with BMI < 25 (*n* = 19) was tested in a supervised manner (Student’s *t* test with a significance threshold of *p* < .05; both groups had comparable age distributions and gender ratios). This post hoc analysis confirmed the associations observed in the TDA analysis, with 65% of the lipids following the same trend (data not shown). However, although the median values differed, the BMI ranges of both groups were similar, and direct correlations between individual lipid concentrations and BMI did not reach significance (linear regression at *p* < .05).

Numbers of macrophages and neutrophils, the two most abundant cell types in ELF, did not differ between the groups, but lymphocyte numbers were significantly (*p* = .013) elevated in group B, whereas squamous epithelial cells were significantly (*p* = .001) higher in group A (Table 2; Supplementary Material 1, Fig. S2).

## Discussion

This study shows that ELF, as sampled by sputum induction, has a diverse lipidome comprising a range of different lipid molecular classes, including glycerophospholipids (PC, PE, PG, PS and PI), sphingolipids (SM, Cer and HexCer) sterol lipids (cholesterol, CE) and glycerolipids (DG, TG). Although this was a cross-sectional study of healthy, non-smoking individuals, topological data analysis identified two distinct sputum lipid profiles. These were differentiated by enrichments of sterols, glycolipids and sphingolipids in one participant group (A), and elevated total lipid concentrations with enrichments of diacyl-PC, PG and PI species in the other (B). Of the various demographic and physiological measurements examined, only BMI was significantly different between these two lipid phenotypes.

### Sputum lipid composition and sample dilution

The overall ELF lipid profile reported here reflects the unique composition of pulmonary surfactant and is broadly similar to previous reports (Dushianthan et al. 2012; Sahu and Lynn 1978; t’Kindt et al. 2015). Unlike human plasma (Quehenberger and Dennis 2011) for example, the ELF lipidome is dominated by a restricted number of mostly di-saturated PC species, including the highly surface-active lipid DPPC (Fessler and Summer 2016; Goss et al. 2012). LC–MS/MS fragmentation confirmed the identities of 95% of sputum lipidome (Table 3), but future studies may require more extensive and detailed structural assignments, particularly of the remaining 5% of low-abundance lipid species.

Although the sputum induction process primarily targets the bronchial ELF, passage of the expectorated sample through the oral cavity leads to a variable degree of mixing with saliva and squamous cells from the oral epithelium and nasopharynx (Fig. 1). Saliva itself is lipid-poor and contains only small amounts of sterols and glycerolipids (DG, TG) (Larsson et al. 1996). As a consequence, the main effect of increasing the amount of saliva in an expectorated sputum sample is dilution of ELF resulting in lower lipid concentration. The abundance of squamous epithelial cells relative to that of other cells is often used as a dilution marker, since they make up > 98% of cells in saliva (Belda et al. 2000; Spanevello et al. 1998, 2000). However, direct comparisons between the number of squamous cells in a sputum sample and concentrations of lung-specific molecular biomarkers have found only weak associations with a large margin of error (Boorsma 2000; Simpson et al. 2004). Other contributors to sputum samples are the membrane fragments and exosomes from immune cells, particularly neutrophils and macrophages. These have distinctive membrane phospholipid profiles (e.g. Postle et al. 2004) which can be used to determine the contribution of cellular material to the overall lipidomic profile (Todd et al. 2010). Sputum lipid group A was characterised by lower total lipid concentrations and an enrichment of sterols, glycolipids and sphingolipids compared to group B. Together with the higher squamous cell numbers, this could suggest a stronger salivary dilution of the samples that were taken from these participants. Therefore, it was important to establish whether the observed TDA grouping was driven by straightforward sample dilution, rather than biological or lifestyle differences. A direct comparison of DPPC concentrations and squamous cell numbers (as per Boorsma 2000) did not show any significant correlation (linear regression, *R*^2^ = 0.210). Moreover, relative abundances of DPPC to other PC molecular species such as PC[30:0], PC[32:1] or PC[36:2], were significantly different between the two groups (Fig. 3). Since saliva would dilute the different surfactant phospholipid species in a uniform manner, we conclude that the differences between the two groups did not result from sample dilution during the induction or subsequent processing.

### Potential effects of weight status on lung lipid metabolism

The different composition and increased sputum lipid concentrations in the participant group with higher BMI (B) raises the intriguing possibility that being overweight affects ELF lipid homeostasis. The impact of diet on lung lipid metabolism and the wider pulmonary system has thus far received very little attention. All pulmonary surfactant phospholipids are synthesised by lung alveolar epithelial type II (AT2) cells, using fatty acids produced either by the fatty acid synthase (FAS) complex in the same cells, or derived from FAS or stored TG reserves in adjacent lipofibroblasts (Bernhard et al. 1997; Brandsma and Postle 2017). However, the degree to which the composition of secreted complex lipids is controlled by the AT2 cells, and to what extent it is driven by the availability of different fatty acids in the circulation remains unclear. The active exchange of lipids between lungs, circulation and liver in a ‘hepato-pulmonary rheostat’ is well documented (Brandsma and Postle 2017; Hunt et al. 2017; Trapnell and Bridges 2017; Zhou et al. 2004), but in vivo evidence is scarce and mostly limited to observations in rodent models. Dietary lipid nutrition can modify pulmonary surfactant composition, shown for example by the temporary enrichment of myristic acid-containing PC species in nursing rat pups feeding on rat milk that is enriched in myristate (Bernhard et al. 2007). More recently, significant dysregulation of lung lipid metabolism was observed in mice fed obesogenic diets (Showalter et al. 2018). The so-called ‘fatty lung’ is at the extreme end of this spectrum, described in rat models of genetically or diet-induced obesity. This phenotype is characterised by increased lung weight, volume and alveolar surface area, but decreased lung compliance (Foster et al. 2010; Inselman et al. 2004) and an impaired response to chronic hypoxia (Yilmaz et al. 2015). Their lung tissue shows a substantial accumulation of TG-filled lipid droplets, as well as collagen deposition, similar to that observed in the livers of non-alcoholic fatty liver disease patients (Byrne and Targher 2015). In addition, the AT2 cell lamellar bodies are enlarged and more abundant than in normal-weight animals, surfactant protein expression and secreted concentrations increased, and lipid-laden ‘foamy’ macrophages are present (Foster et al. 2010; Inselman et al. 2004). Secreted lipid concentrations were also found to be higher, although lipid synthetic rates were not assessed directly (for example as per Brandsma et al. 2017; Postle and Hunt 2009) and no compositional information is available.

It is reasonable to assume that similar processes operate in humans, where they may likewise result in metabolic dysregulation of the lung. Thus, we speculate that the elevated sputum lipid levels and atypical composition in group B may have been driven by the participants’ overall higher weight status. However, a number of limitations of the present study need to be acknowledged. Firstly, samples were acquired from a study not originally designed to investigate the effects of weight status on lipid metabolism. Consequently, weight status was primarily assessed by BMI, which is widely accepted to be a relatively poor predictor of adiposity (Abad and Pallan 2018; Shah and Braverman 2012). Moreover, severely obese participants were excluded from the study, and no effort was made to balance numbers of volunteers in the different weight status groups. Second, sputum induction is a difficult procedure to perform, with success rates between 30 and 40% even in specialised research centres (Chanez et al. 2002). Generation of good quality sputum samples is particularly challenging from healthy individuals (e.g. Lins 2016), since not only are they less likely to volunteer for the procedure, but also, as their airways are not inflamed, they do not have mucus hypersecretion. Therefore, although ours is the largest study of its kind in healthy individuals to date, validation of the findings is required in a larger, and ideally more ethnically diverse cohort. This would not only enable more rigorous statistical testing of the results (e.g. including adjustments for multiple comparisons), but also provide the opportunity for a more targeted study design in terms of weight status (e.g. including underweight and very obese groups and/or patients with diabetes) and better physiological measurements.

### Implications and outlook

Obesity is widely recognised as a contributing factor in the pathophysiology and development of a number of respiratory diseases, including asthma, COPD and pulmonary fibrosis (McDonald et al. 2016; Muc et al. 2016; Romero et al. 2015), a process which may already start in utero (Heerwagen et al. 2010). It is known to affect a range of internal organs (Rutkowski et al. 2015; Sun et al. 2013) and having altered lung lipid metabolism or even a ‘fatty lung’ may be yet another feature associated with being significantly overweight or obese. How AT2 cells would respond to a systemic oversupply of nutrients and fats remains to be elucidated. An excess lipid supply could either be secreted basolaterally back into the circulation, processed and secreted into the alveoli as pulmonary surfactant, or stored in intracellular lipid droplets (Brandsma and Postle 2017). The latter two potentially have significant negative health effects. Redirecting increased lipid influx to intracellular storage rather than adipose tissue (ectopic lipid deposition) is known to induce both pro-inflammatory and pro-fibrotic responses (Ertunc and Hotamisligil 2016). Excess pulmonary surfactant secreted into the airways would normally be cleared by alveolar macrophages, but evidence suggests that oxidation of this material can induce a shift towards a pro-inflammatory phenotype in these cells (Romero et al. 2015). Furthermore, the function of pulmonary surfactant itself depends strongly upon its unique lipid and protein compositions (Burg et al. 2018; Lopez-Rodriguez and Pérez-Gil 2014), and any alterations may affect its ability to lower surface tension at the air/liquid interface within the alveoli, as well as its efficacy as a physical and immunological barrier to the outside environment. In conclusion, there is significant potential for nutrition and weight status to affect lung lipid metabolism, and this may have significantly implications for respiratory health, inflammatory state, and disease risk and severity.

This study used untargeted shotgun mass spectrometry to analyse the lipid composition of the ELF of healthy non-smoking adults acquired through sputum induction, and examined its variability in respect of common demographic variations. Rather than finding a uniform lipidome in this cross-section of ‘normal’ individuals, TDA identified two distinct groups of participants with significantly different sputum lipid compositions. The only demographic difference between these two groups was in BMI, suggesting weight status may be related to differences in lung lipid metabolism, as has been observed in animal models. Because this was a cross-sectional study of healthy individuals, the clinical relevance of such metabolic alterations remain unclear. Further research is needed to confirm the existence of this phenotype in other human cohorts, and to obtain a mechanistic understanding of the relationships between nutrition, obesity and lung lipid metabolism, as well as its clinical implications for pulmonary diseases.

## Electronic supplementary material

Below is the link to the electronic supplementary material.

Supplementary material 1 (DOCX 873 KB)

Supplementary material 2 (PDF 135 KB)

Supplementary material 3 (PDF 160 KB)
