# Supplementary material for: Lipid phenotyping of lung epithelial lining fluid in healthy human volunteers
Source: Metabolomics. 2018 Sep 17;14(10):123. doi: 10.1007/s11306-018-1412-2 (PMC6153688; doi:10.1007/s11306-018-1412-2)

#### **supplementary MATERIAL 1**

#### **Lipid phenotyping of lung epithelial lining fluid in healthy human volunteers**

#### **1 The U-BIOPRED study design**

#### The U-BIOPRED study (Unbiased Biomarkers for the Prediction of Respiratory Disease Outcomes) is a pan-European multi-centre public-private collaboration aimed at (sub)phenotyping patients with severe refractory asthma using a multi-omics systems biology approach (Auffray et al. 2010; Bel et al. 2011; Wheelock et al. 2013). The clinical study itself, which included well-controlled mild to moderate asthmatics and healthy participants as controls, has been described previously (Shaw et al. 2015) and its protocols were approved by all the local Ethics Review Boards. The U-BIOPRED study was performed in 14 clinical centres across Europe with extensive experience in the induction and processing of sputum samples. Participants gave their written informed consent for in-depth characterisation using routine clinical protocols, and haematological and biochemistry blood tests (reported in detail by Shaw and co-workers (2015)), as well as molecular characterisation by a variety of ‘omics platforms. Processed biological samples from all clinical sites were stored in a central biobank (CIGMR Biobank, University of Manchester) where their identifiers were blinded. Induced sputum samples were shipped to the analytical sites in three separate batches and lipidomic profiles were obtained as described below. Identity of the samples was only un-blinded after all the ‘omics analyses and data processing and quality control (QC) steps had been completed.

#### **2 Induced sputum collection and preparation**

Expectorated sputum was collected over a period of up to four times 5 minutes, following inhalation of a nebulized 4.5% hypertonic saline solution and an initial administration of 400 µg Salbutamol (Djukanović et al. 2002), using a standardised method for all subjects (Bafadhel et al. 2011). The mucoid portions of the sputum were collected using forceps, weighed, and solubilised in a solution of 6.8 mM dithioerythritol in HEPES-buffered saline at a 4:1 weight-to-volume ratio. After the non-solubilised mucus was removed by filtration (100 µm), the sputum supernatants were centrifuged twice at 4°C (once at 400x*g* and once at 12,000x*g*) to remove any mucus and bacteria, and finally snap-frozen and stored at -80°C. The sputum cell pellets were processed for quantification by rapid Romanowsky staining (Diff-Quik); eosinophil, neutrophil, macrophage and lymphocyte levels are reported as percentages of total inflammatory cells, while epithelial squamous cells are reported as a percentage of total cell counts. It should be noted that the U-BIOPRED study used a sputum sample quality control limit of 40% squamous cells; any samples exceeding this limit were biobanked, but not made available for further analysis.

#### **3 Lipid analysis**

**3.1 Lipid extraction**

Frozen samples were first thawed to room temperature, before taking a 100 µl aliquot for liquid-liquid extraction. A semi-automated Bligh-Dyer extraction protocol (Bligh and Dyer 1959) on a robotic liquid handling platform (Freedom EVO 100; TECAN, Männedorf, Switzerland) was used. Initially, each sample was made up to a volume of 800 µl with a 0.9% saline solution, before adding 2 ml of methanol (MeOH) and 1 ml of dichloromethane (DCM). In addition, anti-oxidant (10 µl of 5 mg ml^-1^ butylated hydroxytoluene in MeOH) and a mixture of synthetic lipid standards for internal quantification were added (see below). Samples were then mixed vigorously and centrifuged at 3000 rpm for 10 min at 10ºC to remove precipitated proteins. The supernatants were transferred into fresh centrifuge tubes and a further 1 ml of DCM and 1 ml of ultrapure water were added. Following a second mixing and centrifugation step at 3000 rpm for 5 min at 10°C, 1.6 ml of the lower organic phase (equalling ~80%) was recovered. This was split into two aliquots of 800 µl each and dried down under a stream of N_2_ gas (Ultravap RC; Porvair Sciences, Leatherhead, UK). Dried extracts were stored at -80ºC until mass spectrometry analysis.

The internal standard mixture was made up in MeOH:DCM:H_2_O (65:35:8 v/v) and contained the following per 100 µl of sputum: 1 nmol of *1,2-dimyristoyl-sn-glycero-3-phosphocholine* (PC[14:0/14:0]); 1 nmol of *1,2-ditetracosanoyl-sn-glycero-3-phosphocholine* (PC[24:0/24:0]); 400 pmol of *1,2-dimyristoyl-sn-glycero-3-phosphoethanolamine* (PE[14:0/14:0]); 200 pmol of *1,2-dimyristoyl-sn-glycero-3-phosphoglycerol* (PG[14:0/14:0]); 200 pmol of *1,2-dimyristoyl-sn-glycero-3-phosphoserine* (PS[14:0/14:0]); 120 pmol of *1,2-dihexadecanoyl-sn-glycero-3-phosphoinositol* (PI[16:0/16:0]); 100 pmol of *1,2-dimyristoyl-sn-glycero-3-phosphate* (PA[14:0/14:0]); 100 pmol of *1-heptadecanoyl-sn-glycero-3-phosphocholine* (LPC[17:0]); 100 pmol of *1-myristoyl-sn-glycero-3-phosphoethanolamine* (PE[14:0]); 100 pmol of *1-myristoyl-sn-glycero-3-phosphoglycerol* (PG[14:0]); and 100 pmol of *1-myristoyl-sn-glycero-3-phosphate* (PA[14:0]). All standards were obtained specifically for this study from Avanti polar lipids (Alabaster AL, USA).

**3.2 Lipid analysis by direct infusion MS**

Before analysis each sample was reconstituted in 1 ml of MeOH:DCM:50 mM aqueous NH_4_HCO_2_ (65:35:8 v/v), and 20-µl aliquots were removed from each sample and pooled to create a quality control (QC) sample. All measurements were performed on a MaXis 3G ultra-high resolution quadrupole time-of-flight (UHR Q-ToF) mass spectrometer equipped with an electrospray ionization (ESI) source (Bruker Daltonics, Billerica MA, USA), coupled to an UltiMate 3000 ultra-high performance liquid chromatography system (UHPLC; Dionex, Sunnyvale CA, USA). For the initial screening, samples were introduced by loop injection (10 µl) into a continuous stream of MeOH (HPLC grade). Mass spectra were acquired in full scan mode over an *m/*z range of 350-1200 for a period of 3 minutes per injection, which covered the elution time of the sample peak and a 1 min washout period (separate injections for positive and negative ionisation). Each injection was preceded by a 1 min pre-run of MeOH to reduce the risk of sample carry-over, and blank injections were performed after every 4 samples (no significant carry-over was detected). In addition, the pooled QC sample was run after every 4 samples to check for changes in the instrument performance.

**3.3 Lipid identification by UPLC-MS/MS**

Fragmentation analysis for lipid identification was performed using the same instrumental setup. Samples (10 µl) were first separated on a C8 column (Waters Acquity UPLC CSH C8, 130Å, 1.7µm, 2.1mm x 100mm) using mobile phases of: A) MeOH with 50 mM NH_4_HCO_2_ and 0.2% formic acid, and B) 50 mM aqueous NH_4_HCO_2_ with 0.2% formic acid (all LC grade). The following gradient was used: linear increase from 80-98% A at 0.3 ml min^-1^ over the first 10 min, then a linear increase to 100% A at 0.3 ml min^-1^ over the next 10 min, isocratic at 100% A for 25 min but with an increased flow rate of 0.4 ml min^-1^, rapid return to the starting conditions (80% A at 0.3 ml min^-1^) to re-equilibrate the system for 5 min. Data-independent product ion scans were acquired over the entire 50 min gradient using the bbCID (broadband Collision Induced Dissociation) function in Compass (Bruker Daltonics). In this setting the MS rapidly alternates between low and high collision energy without mass selection, resulting in parallel sets of intact precursor and fragment ions over the whole *m/z* range of 350-1200. Precursor and fragment ions were matched retrospectively by their LC retention time and using the well-established fragmentation rules for lipids (Hsu and Turk 2003) to provide confirmation of identities where possible.

**3.4 Mass spectra processing and alignment**

Manipulation of the mass spectra and export of the data were automated in Compass DataAnalysis V4.0 using the attached VBA Method Editor V1.0 (Bruker Daltonics). All screening spectra were first Gaussian smoothed and automated peak picking was performed using a minimum signal threshold of 10 counts. Lock mass calibration of the internal standard peaks was used to align all spectra to within <1 ppm. Spectral data were exported in a mass list format of *m/z* value (peak apex) and intensity (counts). Two separate mass lists were generated for each sample: a combined average over the first and final 10 seconds of the run (the “background” spectrum) and an average over the first 2 minutes of the run (the “sample” spectrum).

To align the large amount of spectral data a hierarchical clustering-based algorithm was used; this was a simplified version of the method developed by Yang (2016) for multidimensional UPLC-IM-MS^E^ data. In short, the data were first divided into bins based on their mass-to-charge ratio (*m/z*). In order to avoid boundary effects due to arbitrary splitting, pre-set bins of 0.05 *m/z* were used which overlapped by 0.025 *m/z* on either side. For example, adjacent bins would be 700.000-700.050 *m/z*, 700.025-700.075 *m/z*, 700.050-700.100 *m/z*, etc. Thus, each ion appears twice in two neighbouring bins. Following this, the ions were aligned within each bin based on the Euclidian distance between every ion pair. The distance between any two ions within the same spectrum was assigned to infinity to avoid grouping together ions originating from the same sample. An average hierarchical clustering analysis was then performed on the distance values, resulting in clusters of ions with similar *m/z* values. Finally, all the overlapping bins were checked for ion clusters that straddled consecutive bins, as this would result in such an ion being split up in the final alignment file. The algorithm automatically resolves such cases by combining the two halves of the ion cluster in one bin and removing it from the other (see Yang 2016 for details and examples).

After alignment, individual “background” spectra were subtracted from the associated “sample” spectra, with negative values changed to zero. Subsequently, an average spectrum from all of the blank sample runs was calculated, and this was also subtracted from each of the “sample” spectra. In this way all possible background signals from the instrument or introduced during sample preparation and storage were excluded from the results. For each sample the average number of counts of each ion were then calculated based on the triplicate injections, with ions present in only one out of three runs removed from the list. Repeatability of the measurements was high, with a coefficient of variation of less than 3% across all ions.

**3.5 Feature selection, quality control and data normalisation**

A combined total of 10235 individual ions (6377 positive and 3858 negative) were detected in the samples. However, around 80% of these ions were present in only one or a few of the samples, but absent or below the limit of detection in the rest. Only 32 ions were consistently detected in all samples, 141 ions in 90% or more of the samples, 214 ions in 80% or more, 291 ions in 60% or more, etcetera in a sigmoidal fashion. Plotting the detection rate versus the abundance of each individual ion showed that the two were unrelated. This distribution presents something of a dilemma in terms of feature selection, since it is unclear which of these low-abundance ions are actual lipids of interest, and which are simply the introduced contaminants and ‘random noise’ seen in any untargeted MS analysis. Large amounts of “missing data” are also problematic for statistical analyses, which often require imputation of zero values, and for batch effect correction programmes such as the widely used R script “ComBat” (Johnson et al. 2007). Hence, a cut-off of 60% detection rate was selected based on the inflection point of the curve, which strikes a balance between inclusivity and statistical rigour. All ions with a lower rate of detection were eliminated from the final data set, irrespective of their abundance.

To account for changes in instrument performance over time, the pooled QC runs were examined for significant shifts (>10%) in signal. One such shift was observed when an emergency shutdown resulted in change in the ratio between positive and negative ionisation. To control for the introduction of batch effects due to instrument performance and known differences in sample work-up dates, all results were processed through ComBat using log transformed data and imputation of a small constant value (1/5 of the smallest overall value) for any missing data.

After QC all ions were normalised to the signal intensity of the DMPC internal standard and the original sample volume to obtain semi-quantitative data (µM relative to DMPC). This step assumes comparable ionisation potential within the mass spectrometer, and a comparison between DMPE and DMPC internal standard signals across all the samples showed no significant ion suppression effects between the two. Since biofluids, and particularly induced sputum, are subject to variable dilution of analytes during sampling and subsequent workup, the MS data were also represented as a fraction of the amount of dipalmitoyl-phosphatidylcholine (DPPC) in each of the samples. This lipid is a biomarker for pulmonary surfactant and was therefore chosen analogous to a ‘housekeeping’ gene or protein (see discussion in the main text).

#### **4 Topological clustering and differential feature analysis**

Topological data analysis (TDA) is an unsupervised method for representing highly complex data in a structured 3-dimensional network of nodes (which comprise two or more data points that are similar to each other in multiple dimensions) and edges (lines connecting nodes with at least one shared data point), which retains the geometric ‘shape’ of the data (Carlsson 2009). Sub-groups within the dataset show up as discrete clusters or flares of highly interconnected nodes and can be selected for further statistical testing using conventional methods (Bigler et al. 2016; Hinks et al. 2016). For this study, TDA was used to visualise clusters of participants within the healthy non-smoking adults with comparable sputum lipid profiles (n=41). Analyses were performed using the Ayasdi machine intelligence platform (Ayasdi, Palo Alto, CA, USA).

TDA was performed on the selected lipid data set (291 ions) using both semi-quantitative data and abundances normalised to the amount of DPPC in each sample. For each TDA a normalised correlation metric was chosen to negate the large dynamic range of the data, and this was combined with multidimensional scaling (MDS) lenses at resolution and gains settings appropriate to the size of the data set (see figure legends). Discreet clusters of participants were defined manually within the TDA networks (Bigler et al. 2016; Hinks et al. 2016). Finally, differences in lipid composition and participant metadata (such as age, gender, BMI, cell counts) between each of the selected subgroups were examined using the Mann-Whitney U test with a significance threshold of p<0.05. No adjustments for multiple testing were done because of the relatively small cohort size, and validation of the results in follow-up studies is therefore warranted.

**References**

Auffray, C., Adcock, I. A., Chung, K. F., Djukanović, R., Pison, C., & Sterk, P. J. (2010) An integrative systems biology approach to understanding pulmonary diseases. Chest, 137(6): 1410-1416. <https://doi.org/10.1378/chest.09-1850>.

Bafadhel, M., McCormick, M., Saha, S., McKenna, S., Shelley, M., Hargadon, B., et al. (2011) Profiling of sputum inflammatory mediators in asthma and chronic obstructive pulmonary disease. Respiration, 83: 36-44. <https://doi.org/10.1159/000330667>.

Bel, E. H., Sousa, A. R., Fleming, L., Bush, A., Chung, K. F., Versnel, J., et al. (2011) Diagnosis and definition of severe refractory asthma: an international consensus statement from the Innovative Medicine Initiative (IMI). Thorax, 66(10): 910-917. <https://doi.org/10.1136/thx.2010.153643>.

Bigler, J., Boedigheimer, M., Schofield, J. P. R., Skipp, P. J., Corfield, J., Rowe, A., et al. (2016). A severe asthma disease signature from gene expression profiling of peripheral blood from UBIOPRED cohorts. American Journal of Respiratory and Critical Care Medicine, 195(10), 1311-1320. <https://doi.org/10.1164/rccm.201604-0866OC>.

Bligh, E. G., & Dyer, W. J. (1959). A rapid method of total lipid extraction and purification. Canadian Journal of Biochemistry and Physiology, 37(8), 911-917. <https://doi.org/10.1139/o59-099>.

Carlsson, G. (2009) Topology and data. Bulletin of the American Mathematical Society, 46(2): 255–308. <https://doi.org/10.1090/S0273-0979-09-01249-X>.

Djukanović, R., Sterk, P. J., Fahy, J. V., & Hargreave, F. E. (2002) Standardised methodology of sputum induction and processing. European Respiratory Journal, 20(37 suppl): 1s-2s. <https://doi.org/10.1183/09031936.02.00000102>.

Hinks, T. S. C., Brown, T., Lau, L. C. K., Rupani, H., Barber, C., Elliott, S., et al. (2016). Multidimensional endotyping in patients with severe asthma reveals inflammatory heterogeneity in matrix metalloproteinases and chitinase 3–like protein 1. The Journal of Allergy and Clinical Immunology, 138(1), 61-75. <https://doi.org/10.1016/j.jaci.2015.11.020>.

Hsu, F. F., & Turk, J. (2003). Electrospray ionization/tandem quadrupole mass spectrometric studies on phosphatidylcholines: the fragmentation processes. Journal of American Society for Mass Spectrometry, 14(4), 352-363. <https://doi.org/10.1016/S1044-0305(03)00064-3>.

Johnson, W. E., Li, C., & Rabinovic, A. (2007) Adjusting batch effects in microarray expression data using empirical Bayes methods. Biostatistics, 8(1), 118-127. <https://doi.org/10.1093/biostatistics/kxj037>.

Shaw, D. E., Sousa, A. R., Fowler, S. J., Fleming, L. J., Roberts, G., Corfield, J., et al. (2015). Clinical and inflammatory characteristics of the European U-BIOPRED adult severe asthma cohort. European Respiratory Journal, 46(5), 1308-1321. <https://doi.org/10.1183/13993003.50779-2015>.

Wheelock, C. E., Goss, V. M., Balgoma, D., Nicholas, B., Brandsma, J., Skipp, P. J., et al. (2013) Application of 'omics technologies to biomarker discovery in inflammatory lung diseases. European Respiratory Journal, 42(3): 802-825. <https://doi.org/10.1183/09031936.00078812>.

Yang, X. (2016). Analysing datafied life. PhD Thesis, Imperial College London, pp. 288. <https://spiral.imperial.ac.uk/handle/10044/1/33722>.

**Supplementary figures**

Fig. S1: TDA networks of lipid concentrations show a consistent presence of two groups within the healthy non-smoking sputum sample set (the small intermediate cluster has low persistence and joins either group A or B at slightly altered resolution or gain settings). TDA was performed on 291 ions and use a normalised correlation metric and two MDS lenses. The network is coloured by the total amount of lipid measured (average value of the samples in each node), with blue indicating low, and red high concentrations. The figure was obtained with the Ayasdi machine intelligence platform (www.ayasdi.com/platform).

Fig. S2: Comparison of differential cell counts between the two TDA groups showed significant differences in lymphocytes (higher in group B) and squamous epithelial cells (higher in group A), but not macrophages, neutrophils or eosinophils.


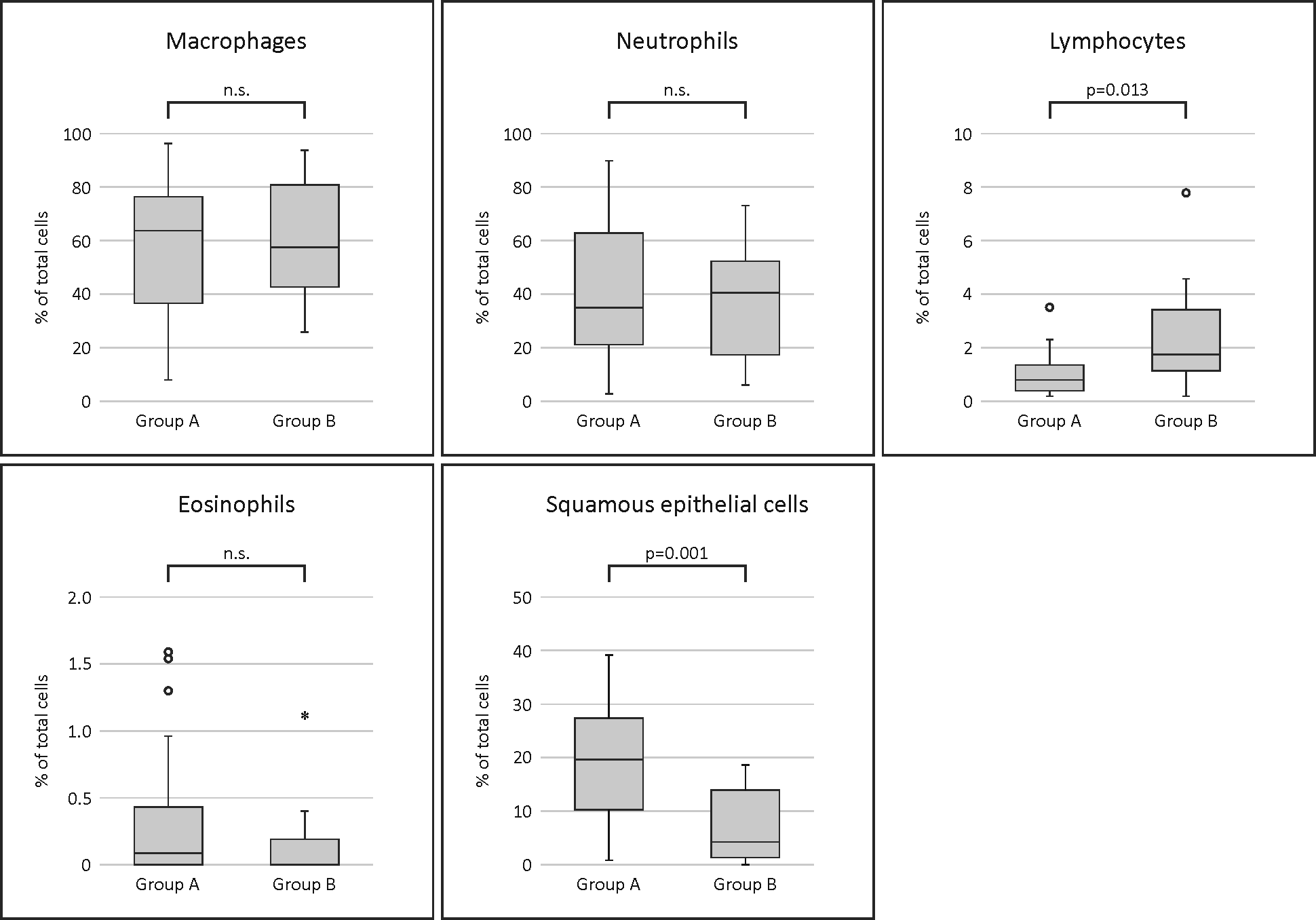

Supplement: Supplementary file 1 — Supplementary material 1 (DOCX 873 KB) [file 11306_2018_1412_MOESM1_ESM.docx]
