# Supplementary material for: Lipid phenotyping of lung epithelial lining fluid in healthy human volunteers
Source: Metabolomics. 2018 Sep 17;14(10):123. doi: 10.1007/s11306-018-1412-2 (PMC6153688; doi:10.1007/s11306-018-1412-2)
Supplement: Supplementary file 2 — Supplementary material 2 (PDF 135 KB) [file 11306_2018_1412_MOESM2_ESM.pdf]

SUPPLEMENTARY MATERIAL 2

Lipid phenotyping of lung epithelial lining fluid in healthy human volunteers

Supplementary Table:

A total of 291 ions were measured in 60% or more of the sputum samples; listed here are their assignment and the associated ions that were detected (electrospray ionization mode, *m/z* values, mass isotopomers and adducts). The table also shows the abundances (median and range) of each lipid, either normalised to DPPC or as semi-quantitative data, plus the differences between TDA groups A and B and whether these differences were statistically significant (Mann-Whitney U test with a threshold of p<0.05). DPPC itself was normalised to the total amount of lipid in the samples. Where significant, the p-value is given and the abundance cells colour coded, with red signifying lower median values and green higher ones

Note that the direct infusion shotgun method used in this study does not allow for discrimination between, and quantification of, isomeric or isobaric lipid species. So for example, the single ion at *m/z* 790.563 could be either a PC (with 36 carbon atoms and no double bonds in the fatty acyl chains) or a PE (with 40 carbon atoms and 7 double bonds in the fatty acyl chains), or indeed comprise a mixture of PCs with different fatty acyl combinations (e.g. PC[18:0/18:0], PC[16:0/20:0], PC[14:0/22:0], etc.). By performing LC-MS/MS on the pooled QC sample the presence of both PC[36:0] and PE[40:7] was confirmed, and the *m/z* 790.563 ion is therefore listed twice in the table but highlighted with an asterisk (*). However, for quantitative purposes and in the topological data analysis all isomeric and isobaric lipids were treated as a single variable.

Lipid					Relative abundance (% of DPPC)				Abundance (µM normalised to DMPC internal standard)			
Assignment	ESI	m/z	Mass isotopomer	Ion	All samples median [range]	MW U test p-value	Group A median [range]	Group B median [range]	All samples median [range]	MW U test p-value	Group A median [range]	Group B median [range]
**Glycerophospholipids**												
LPC[16:1]	+ve	494.325	M+0	[M+H]+	0.08 [0-0.25]	n.s.	0.07 [0-0.25]	0.10 [0-0.20]	0.04 [0-0.60]	p<0.001	0.02 [0-0.13]	0.20 [0-0.60]
LPC[16:0]	+ve	496.340	M+0	[M+H]+	0.74 [0.21-5.69]	n.s.	0.70 [0.21-5.69]	0.80 [0.26-2.28]	0.34 [0.01-5.26]	p<0.001	0.28 [0.01-1.22]	1.18 [0.20-5.26]
	+ve	497.344	M+1	[M+H]+								
LPC[18:3]	+ve	518.324	M+0	[M+H]+	0.17 [0-0.96]	n.s.	0.19 [0-0.96]	0.13 [0.03-0.53]	0.07 [0-1.54]	p<0.001	0.05 [0-0.26]	0.24 [0.02-1.54]
	+ve	519.329	M+1	[M+H]+								
LPC[18:2]	+ve	520.337	M+0	[M+H]+	0.15 [0-1.08]	n.s.	0.16 [0-1.08]	0.12 [0.03-0.47]	0.06 [0-1.61]	p=0.001	0.04 [0-0.35]	0.12 [0.03-1.61]
	+ve	521.341	M+1	[M+H]+								
LPC[18:1]	+ve	522.354	M+0	[M+H]+	0.23 [0.06-0.96]	n.s.	0.23 [0.06-0.96]	0.22 [0.06-0.75]	0.09 [0.02-1.25]	p<0.001	0.07 [0.02-0.21]	0.28 [0.05-1.25]
	+ve	523.352	M+1	[M+H]+								
LPC[18:0]	+ve	524.367	M+0	[M+H]+	0.15 [0-0.92]	n.s.	0.19 [0-0.92]	0.13 [0-0.47]	0.06 [0-0.92]	p=0.002	0.05 [0-0.21]	0.16 [0-0.92]
	+ve	525.383	M+1	[M+H]+								
LPC[24:0]	+ve	608.386	M+0	[M+H]+	0.09 [0-1.49]	n.s.	0.09 [0-1.49]	0.13 [0-0.54]	0.05 [0-2.12]	p=0.002	0.02 [0-0.44]	0.19 [0-2.12]
PCa[30:0]	+ve	692.532*	M+0	[M+H]+	0.19 [0-0.38]	p=0.030	0.21 [0-0.38]	0.15 [0.09-0.29]	0.11 [0-0.83]	p=0.001	0.10 [0-0.14]	0.26 [0.07-0.83]
	+ve	693.537*	M+1	[M+H]+								
PC[30:1]	+ve	704.564*	M+0	[M+H]+	0.41 [0.18-2.45]	p=0.044	0.49 [0.18-2.45]	0.35 [0.20-0.82]	0.18 [0.04-1.83]	p<0.001	0.15 [0.04-0.41]	0.50 [0.14-1.83]
	+ve	705.578*	M+1	[M+H]+								
PC[30:0]	+ve	706.539	M+0	[M+H]+	11.39 [5.00-20.07]	p=0.040	10.53 [5.00-20.07]	12.88 [7.20-18.68]	5.73 [0.21-65.88]	p<0.001	3.66 [0.21-11.20]	21.24 [5.12-65.88]
	+ve	707.542	M+1	[M+H]+								
	+ve	708.543	M+2	[M+H]+								
	+ve	709.543	M+3	[M+H]+								
	-ve	750.532	M+0	[M+HCOO]-								
	-ve	751.535	M+1	[M+HCOO]-								
PCa[32:2]	+ve	716.543*	M+0	[M+H]+	0.13 [0.06-0.54]	p=0.007	0.14 [0.07-0.54]	0.09 [0.06-0.19]	0.06 [0.01-0.69]	p<0.001	0.04 [0.01-0.10]	0.17 [0.05-0.69]
	+ve	717.553*	M+1	[M+H]+								
PCa[32:1]	+ve	718.558*	M+0	[M+H]+	0.54 [0.35-1.40]	n.s.	0.55 [0.35-1.40]	0.51 [0.36-0.93]	0.28 [0.02-2.09]	p<0.001	0.18 [0.02-0.46]	0.91 [0.25-2.09]
	+ve	719.563*	M+1	[M+H]+								
PCa[32:0]	+ve	720.560*	M+0	[M+H]+	2.53 [1.45-5.14]	n.s.	2.42 [1.45-5.14]	2.61 [1.49-3.98]	1.26 [0.07-13.52]	p<0.001	0.76 [0.07-2.59]	4.89 [1.05-13.52]
	+ve	721.565*	M+1	[M+H]+								
	+ve	722.559	M+2	[M+H]+								
PC[32:2]	+ve	730.550*	M+0	[M+H]+	0.41 [0-0.63]	n.s.	0.36 [0-0.55]	0.44 [0.28-0.63]	0.22 [0-2.38]	p<0.001	0.17 [0-0.49]	0.73 [0.19-2.38]
	+ve	731.603*	M+1	[M+H]+								
PC[32:1]	+ve	732.554*	M+0	[M+H]+	10.93 [5.28-18.68]	p=0.001	9.76 [5.28-14.83]	14.36 [7.49-18.68]	5.55 [0.21-69.42]	p<0.001	3.35 [0.21-14.62]	20.01 [3.47-69.42]
	+ve	733.558*	M+1	[M+H]+								
PC[32:0]	+ve	734.569	M+0	[M+H]+	33.13 [22.53-40.71]†	p=0.26	34.61 [22.53-40.71]†	31.51 [29.00-36.19]†	53.00 [2.44-387.48]	p<0.001	35.77 [2.44-98.28]	147.02 [42.73-387.48]
	+ve	735.573	M+1	[M+H]+								
	+ve	736.575	M+2	[M+H]+								
	+ve	737.577	M+3	[M+H]+								
	+ve	738.573*	M+4	[M+H]+								
	-ve	778.561	M+0	[M+HCOO]-								
	-ve	779.564	M+1	[M+HCOO]-								
	-ve	780.567	M+2	[M+HCOO]-								
PCa[34:2]	+ve	744.564*	M+0	[M+H]+	0.38 [0-0.65]	n.s.	0.34 [0-0.65]	0.42 [0.24-0.50]	0.20 [0-1.80]	p<0.001	0.13 [0-0.33]	0.70 [0.15-1.80]
	+ve	745.568*	M+1	[M+H]+								
PCa[34:1]	+ve	746.579*	M+0	[M+H]+	1.32 [0.73-2.02]	n.s.	1.33 [0.83-2.02]	1.28 [0.73-1.87]	0.62 [0.04-5.93]	p<0.001	0.39 [0.04-1.03]	2.29 [0.42-5.93]
	+ve	747.582*	M+1	[M+H]+								
PCa[34:0]	+ve	748.584*	M+0	[M+H]+	2.26 [1.21-3.41]	n.s.	2.13 [1.21-3.41]	2.40 [1.30-3.32]	1.08 [0.06-12.03]	p<0.001	0.63 [0.06-2.31]	4.28 [0.75-12.03]
	+ve	749.576	M+1	[M+H]+								
	+ve	750.562	M+2	[M+H]+								
	+ve	751.563	M+3	[M+H]+								

Continued

Lipid					Relative abundance (% of DPPC)				Abundance (µM normalised to DMPC internal standard)			
Assignment	ESI	m/z	Mass isotopomer	Ion	All samples median [range]	MW U test p-value	Group A median [range]	Group B median [range]	All samples median [range]	MW U test p-value	Group A median [range]	Group B median [range]
PC[34:4]	+ve	754.540	M+0	[M+H]+	0.84 [0.52-1.68]	p=0.002	0.74 [0.52-1.68]	0.99 [0.71-1.41]	0.40 [0.04-4.15]	p<0.001	0.26 [0.04-0.78]	1.59 [0.40-4.15]
	+ve	755.544	M+1	[M+H]+								
PC[34:3]	+ve	756.553	M+0	[M+H]+	6.55 [4.37-11.47]	p<0.001	5.82 [4.37-10.94]	8.04 [5.41-11.47]	3.71 [0.27-37.24]	p<0.001	2.12 [0.27-6.03]	13.47 [3.82-37.24]
	+ve	757.555	M+1	[M+H]+								
PC[34:2]	+ve	758.568	M+0	[M+H]+	6.89 [3.67-10.93]	p<0.001	5.44 [3.67-8.76]	8.18 [5.70-10.93]	3.81 [0.14-39.46]	p<0.001	1.83 [0.14-6.92]	10.73 [4.49-39.46]
	+ve	759.572	M+1	[M+H]+								
	-ve	802.560	M+0	[M+HCOO]-								
	-ve	804.576	M+1	[M+HCOO]-								
PC[34:1]	+ve	760.585	M+0	[M+H]+	18.10 [10.08-28.09]	p<0.001	15.90 [10.08-22.85]	21.37 [12.21-28.09]	8.72 [0.36-108.85]	p<0.001	5.23 [0.36-17.23]	31.89 [6.80-108.85]
	+ve	761.588	M+1	[M+H]+								
	-ve	805.579	M+0	[M+HCOO]-								
	-ve	806.588	M+1	[M+HCOO]-								
PC[34:0]	+ve	762.596	M+0	[M+H]+	4.61 [3.04-7.62]	p=0.001	4.12 [3.04-6.16]	5.23 [3.19-7.62]	2.20 [0.11-29.54]	p<0.001	1.26 [0.11-4.53]	7.51 [1.78-29.54]
	+ve	763.603	M+1	[M+H]+								
	+ve	764.606*	M+2	[M+H]+								
PCa[36:2]	+ve	772.592	M+0	[M+H]+	0.23 [0-0.34]	p=0.041	0.22 [0-0.32]	0.27 [0-0.34]	0.15 [0-1.16]	p<0.001	0.09 [0-0.18]	0.53 [0-1.16]
PCa[36:1]	+ve	774.605	M+0	[M+H]+	0.34 [0-0.63]	n.s.	0.32 [0.20-0.63]	0.35 [0-0.48]	0.18 [0-1.51]	p<0.001	0.09 [0.02-0.26]	0.62 [0-1.51]
	+ve	775.556	M+1	[M+H]+								
PCa[36:0]	+ve	776.562	M+0	[M+H]+	0.27 [0-0.77]	n.s.	0.38 [0-0.77]	0.26 [0.15-0.50]	0.17 [0-1.39]	p<0.001	0.10 [0-0.30]	0.44 [0.15-1.39]
	+ve	777.564	M+1	[M+H]+								
	+ve	778.581	M+2	[M+H]+								
PC[36:5]	+ve	780.558	M+0	[M+H]+	0.55 [0-1.16]	p<0.001	0.49 [0-1.06]	0.70 [0.50-1.16]	0.35 [0-3.28]	p<0.001	0.17 [0-0.51]	1.12 [0.37-3.28]
	+ve	781.561	M+1	[M+H]+								
PC[36:4]	+ve	782.569	M+0	[M+H]+	2.18 [1.25-3.95]	p<0.001	1.71 [1.25-3.21]	2.60 [2.16-3.95]	1.20 [0.06-13.94]	p<0.001	0.48 [0.06-2.23]	3.90 [1.28-13.94]
PC[36:3]	+ve	784.583	M+0	[M+H]+	1.49 [0-3.21]	p<0.001	1.20 [0-3.21]	1.95 [0.86-2.97]	1.04 [0-11.49]	p<0.001	0.68 [0-2.16]	2.41 [0.48-11.49]
	+ve	785.588	M+1	[M+H]+								
PC[36:2]	+ve	786.599	M+0	[M+H]+	2.53 [1.28-5.00]	p=0.001	2.31 [1.28-4.70]	3.13 [2.17-5.00]	1.36 [0.07-19.37]	p<0.001	0.86 [0.07-2.61]	4.13 [1.21-19.37]
	+ve	787.606	M+1	[M+H]+								
	-ve	830.590	M+0	[M+HCOO]-								
PC[36:1]	+ve	788.613	M+0	[M+H]+	1.52 [0.87-2.99]	n.s.	1.34 [0.87-2.75]	1.72 [0.87-2.99]	0.76 [0.05-11.59]	p<0.001	0.43 [0.05-1.26]	2.47 [0.48-11.59]
	+ve	789.619	M+1	[M+H]+								
PC[36:0]	+ve	790.563*	M+0	[M+H]+	0.59 [0.12-3.60]	n.s.	0.72 [0.12-3.60]	0.45 [0.27-1.14]	0.32 [0.03-3.13]	p<0.001	0.23 [0.03-0.72]	0.78 [0.23-3.13]
	+ve	791.564*	M+1	[M+H]+								
Putative PC	+ve	794.592*	NA	NA	0.35 [0.03-0.61]	n.s.	0.31 [0.03-0.54]	0.40 [0.23-0.61]	0.18 [0.01-2.00]	p<0.001	0.10 [0.01-0.23]	0.60 [0.24-2.00]
Putative PC	+ve	796.601*	NA	NA	0.17 [0-0.73]	n.s.	0.19 [0-0.73]	0.16 [0.10-0.24]	0.09 [0-0.80]	p<0.001	0.06 [0-0.16]	0.27 [0.08-0.80]
PC[38:6]	+ve	806.568	M+0	[M+H]+	0.33 [0-0.67]	p=0.002	0.27 [0-0.65]	0.38 [0.22-0.67]	0.20 [0-2.53]	p<0.001	0.11 [0-0.34]	0.63 [0.12-2.53]
	+ve	807.572	M+1	[M+H]+								
PC[38:5]	+ve	808.582	M+0	[M+H]+	0.48 [0.29-1.09]	p=0.007	0.45 [0.29-1.09]	0.56 [0.43-0.84]	0.25 [0.03-3.01]	p<0.001	0.13 [0.03-0.49]	0.84 [0.25-3.01]
	+ve	809.588	M+1	[M+H]+								
PC[38:4]	+ve	810.597*	M+0	[M+H]+	0.48 [0.28-1.66]	n.s.	0.47 [0.28-1.66]	0.49 [0.36-0.83]	0.24 [0.03-2.93]	p<0.001	0.13 [0.03-0.48]	0.74 [0.22-2.93]
	+ve	811.610*	M+1	[M+H]+								
PC[38:2]	+ve	814.539	M+0	[M+H]+	0.13 [0-0.56]	n.s.	0.21 [0-0.56]	0.12 [0.06-0.50]	0.07 [0-1.17]	p<0.001	0.05 [0-0.20]	0.23 [0.05-1.17]
	+ve	815.539	M+1	[M+H]+								
PC[38:1]	+ve	816.571	M+0	[M+H]+	0.10 [0-0.42]	p=0.003	0.14 [0-0.42]	0.08 [0-0.18]	0.06 [0-0.41]	p<0.001	0.04 [0-0.08]	0.11 [0-0.41]
	+ve	817.582	M+1	[M+H]+								
PC[38:0]	+ve	818.593	M+0	[M+H]+	0.06 [0-0.16]	n.s.	0.08 [0-0.16]	0.06 [0-0.10]	0.04 [0-0.49]	p<0.001	0.03 [0-0.06]	0.09 [0-0.49]
Putative PC	+ve	826.669*	NA	NA	0.05 [0-0.12]	n.s.	0.06 [0-0.12]	0.05 [0-0.06]	0.03 [0-0.39]	p=0.027	0.02 [0-0.07]	0.09 [0-0.39]
	+ve	827.692*	NA	NA								
PCa[40:2]	+ve	828.692*	M+0	[M+H]+	0.08 [0-0.38]	p<0.001	0.14 [0-0.38]	0.05 [0-0.16]	0.04 [0-0.56]	n.s.	0.03 [0-0.12]	0.04 [0-0.56]
	+ve	829.690*	M+1	[M+H]+								

Continued

Lipid					Relative abundance (% of DPPC)				Abundance (µM normalised to DMPC internal standard)			
Assignment	ESI	m/z	Mass isotopomer	Ion	All samples median [range]	MW U test p-value	Group A median [range]	Group B median [range]	All samples median [range]	MW U test p-value	Group A median [range]	Group B median [range]
PCa[40:1]	+ve	830.699*	M+0	[M+H]+	0.05 [0-0.18]	p=0.036	0.06 [0-0.18]	0.04 [0-0.07]	0.02 [0-0.38]	p=0.048	0.02 [0-0.05]	0.09 [0-0.38]
	+ve	831.698*	M+1	[M+H]+								
PC[40:6]	+ve	834.604	M+0	[M+H]+	0.14 [0-0.24]	n.s.	0.17 [0-0.24]	0.11 [0-0.19]	0.08 [0-0.71]	p<0.001	0.07 [0-0.10]	0.23 [0-0.71]
PCa[42:2]	+ve	856.724*	M+0	[M+H]+	0.05 [0-0.14]	p=0.018	0.07 [0-0.14]	0.03 [0-0.13]	0.03 [0-0.25]	n.s.	0.02 [0-0.09]	0.04 [0-0.25]
	+ve	857.709	M+1	[M+H]+								
PC[44:11]	+ve	880.596*	M+0	[M+H]+	0.13 [0-0.27]	n.s.	0.13 [0-0.27]	0.10 [0.06-0.20]	0.06 [0-0.60]	p<0.001	0.04 [0-0.09]	0.16 [0.05-0.60]
	+ve	881.599	M+1	[M+H]+								
PE[32:1]	+ve	690.598	M+0	[M+H]+	0.12 [0-0.47]	p=0.024	0.16 [0-0.47]	0.04 [0-0.12]	0.04 [0-0.11]	p=0.036	0.05 [0-0.11]	0.02 [0-0.02]
PE[32:0]	+ve	692.532*	M+0	[M+H]+	0.19 [0-0.38]	p=0.036	0.21 [0-0.38]	0.15 [0.09-0.29]	0.11 [0-0.83]	p=0.001	0.10 [0-0.14]	0.26 [0.07-0.83]
	+ve	693.537*	M+1	[M+H]+								
PEa[34:2]	+ve	702.533	M+0	[M+H]+	0.14 [0-1.54]	p=0.022	0.24 [0-1.54]	0.12 [0-0.31]	0.06 [0-1.40]	p=0.045	0.05 [0-0.21]	0.17 [0-1.40]
PEa[34:1]	+ve	704.564*	M+1	[M+H]+	0.41 [0.18-2.45]	p=0.046	0.49 [0.18-2.45]	0.35 [0.20-0.82]	0.18 [0.04-1.83]	p<0.001	0.15 [0.04-0.41]	0.50 [0.14-1.83]
PE[34:2]	+ve	716.543*	M+0	[M+H]+	0.13 [0.06-0.54]	p=0.010	0.14 [0.07-0.54]	0.09 [0.06-0.19]	0.06 [0.01-0.69]	p<0.001	0.04 [0.01-0.10]	0.17 [0.05-0.69]
	+ve	717.553*	M+1	[M+H]+								
PE[34:1]	+ve	718.558*	M+0	[M+H]+	0.54 [0.35-1.40]	n.s.	0.55 [0.35-1.40]	0.51 [0.36-0.93]	0.28 [0.02-2.09]	p<0.001	0.18 [0.02-0.46]	0.91 [0.25-2.09]
	+ve	719.563*	M+1	[M+H]+								
PE[34:0]	+ve	720.560*	M+0	[M+H]+	2.53 [1.45-5.14]	n.s.	2.42 [1.45-5.14]	2.61 [1.49-3.98]	1.26 [0.07-13.52]	p<0.001	0.76 [0.07-2.59]	4.89 [1.05-13.52]
	+ve	721.565*	M+1	[M+H]+								
PEa[36:2]	+ve	730.550*	M+0	[M+H]+	0.41 [0-0.63]	n.s.	0.36 [0-0.55]	0.44 [0.28-0.63]	0.22 [0-2.38]	p<0.001	0.17 [0-0.49]	0.73 [0.19-2.38]
	+ve	731.603*	M+1	[M+H]+								
PEa[36:1]	+ve	732.554*	M+0	[M+H]+	10.93 [5.28-18.68]	p=0.002	9.76 [5.28-14.83]	14.36 [7.49-18.68]	5.55 [0.21-69.42]	p<0.001	3.35 [0.21-14.62]	20.01 [3.47-69.42]
	+ve	733.558*	M+1	[M+H]+								
PE[36:5]	+ve	738.573*	M+0	[M+H]+	0.21 [0-0.34]	n.s.	0.21 [0-0.34]	0.21 [0-0.29]	0.12 [0-1.06]	p<0.001	0.08 [0-0.20]	0.40 [0.09-1.06]
	+ve	739.556	M+1	[M+H]+								
PE[36:4]	+ve	740.539	M+0	[M+H]+	0.13 [0-0.38]	n.s.	0.12 [0-0.26]	0.14 [0-0.38]	0.08 [0-0.71]	p<0.001	0.06 [0-0.19]	0.16 [0-0.71]
	+ve	741.542	M+1	[M+H]+								
PE[36:3]	+ve	742.545	M+0	[M+H]+	0.37 [0.21-0.61]	p=0.002	0.28 [0.21-0.61]	0.46 [0.26-0.58]	0.20 [0.01-1.89]	p<0.001	0.09 [0.01-0.26]	0.77 [0.21-1.89]
PE[36:2]	+ve	744.564*	M+0	[M+H]+	0.38 [0-0.65]	n.s.	0.34 [0-0.65]	0.42 [0.24-0.50]	0.20 [0-1.80]	p<0.001	0.13 [0-0.33]	0.70 [0.15-1.80]
	+ve	745.568*	M+1	[M+H]+								
PE[36:1]	+ve	746.579*	M+0	[M+H]+	1.32 [0.73-2.02]	n.s.	1.33 [0.83-2.02]	1.28 [0.73-1.87]	0.62 [0.04-5.93]	p<0.001	0.39 [0.04-1.03]	2.29 [0.42-5.93]
	+ve	747.582*	M+1	[M+H]+								
	+ve	748.584*	M+2	[M+H]+								
PE[38:6]	+ve	764.606*	M+0	[M+H]+	0.78 [0-23.55]	n.s.	0.85 [0-23.55]	0.46 [0.18-3.10]	0.45 [0-2.57]	p<0.001	0.16 [0-1.39]	1.04 [0.17-2.57]
	+ve	765.608	M+1	[M+H]+								
PE[38:5]	+ve	766.559	M+0	[M+H]+	0.44 [0-0.76]	n.s.	0.36 [0-0.76]	0.49 [0.19-0.75]	0.29 [0-2.22]	p<0.001	0.15 [0-0.37]	0.84 [0.29-2.22]
	+ve	767.561	M+1	[M+H]+								
PE[38:4]	+ve	768.565	M+0	[M+H]+	0.37 [0-1.05]	n.s.	0.40 [0-1.05]	0.37 [0.27-0.54]	0.22 [0-1.64]	p<0.001	0.13 [0-0.29]	0.65 [0.18-1.64]
	+ve	769.566	M+1	[M+H]+								
PE[40:7]	+ve	790.563*	M+0	[M+H]+	0.59 [0.12-3.60]	n.s.	0.72 [0.12-3.60]	0.45 [0.27-1.14]	0.32 [0.03-3.13]	p<0.001	0.23 [0.03-0.72]	0.78 [0.23-3.13]
	+ve	791.564*	M+1	[M+H]+								
PE[40:6]	+ve	792.573	M+0	[M+H]+	0.37 [0.05-1.28]	n.s.	0.32 [0.05-1.28]	0.38 [0.20-0.70]	0.24 [0.01-1.73]	p<0.001	0.12 [0.01-0.30]	0.59 [0.24-1.73]
	+ve	793.578	M+1	[M+H]+								
PE[40:5]	+ve	794.592*	M+0	[M+H]+	0.35 [0.03-0.61]	n.s.	0.31 [0.03-0.54]	0.40 [0.23-0.61]	0.18 [0.01-2.00]	p<0.001	0.10 [0.01-0.23]	0.60 [0.24-2.00]
	+ve	795.595	M+1	[M+H]+								
PE[40:4]	+ve	796.601*	M+0	[M+H]+	0.17 [0-0.73]	n.s.	0.19 [0-0.73]	0.16 [0.10-0.24]	0.09 [0-0.80]	p<0.001	0.06 [0-0.16]	0.27 [0.08-0.80]
	+ve	797.528	M+1	[M+H]+								
Putative PE	+ve	810.597*	M+0	NA	0.48 [0.28-1.66]	n.s.	0.47 [0.28-1.66]	0.49 [0.36-0.83]	0.24 [0.03-2.93]	p<0.001	0.13 [0.03-0.48]	0.74 [0.22-2.93]
	+ve	811.610*	M+1	NA								
PS[36:1]	-ve	788.546	M+0	[M-H]-	4.44 [0-28.87]	n.s.	5.95 [0-28.87]	3.73 [0-11.44]	2.61 [0-14.28]	p=0.002	1.80 [0-4.40]	3.65 [0-14.28]
	-ve	789.548	M+1	[M-H]-								

Continued

Lipid					Relative abundance (% of DPPC)				Abundance (µM normalised to DMPC internal standard)			
**Assignment**	**ESI**	**m/z**	**Mass isotopomer**	**Ion**	**All samples median [range]**	**MW U test p-value**	**Group A median [range]**	**Group B median [range]**	**All samples median [range]**	**MW U test p-value**	**Group A median [range]**	**Group B median [range]**
PG[34:1]	-ve	747.529	M+0	[M-H]-	11.07 [0-20.60]	n.s.	9.45 [0-19.29]	11.44 [0-20.60]	4.90 [0-33.11]	p<0.001	3.51 [0-6.05]	10.19 [0-33.11]
	-ve	748.529	M+1	[M-H]-								
PG[36:2]	-ve	773.534	M+0	[M-H]-	6.03 [0-13.65]	p=0.002	5.18 [0-8.80]	6.91 [0-13.65]	2.92 [0-16.91]	p<0.001	1.88 [0-4.36]	5.47 [0-16.91]
	-ve	774.538	M+1	[M-H]-								
PG[36:1]	-ve	775.549	M+0	[M-H]-	8.03 [0-14.90]	n.s.	7.25 [0-13.02]	8.64 [0-14.90]	3.63 [0-20.66]	p<0.001	2.32 [0-4.17]	6.29 [0-20.66]
	-ve	776.547	M+1	[M-H]-								
PI[36:2]	-ve	861.551	M+0	[M-H]-	2.36 [0-5.76]	n.s.	2.18 [0-4.14]	2.59 [0-5.76]	1.62 [0-11.97]	p<0.001	1.10 [0-1.79]	3.38 [0-11.97]
	+ve	863.569	M+0	[M+H]+								
	+ve	880.596*	M+0	[M+NH4]+								
	+ve	885.558	M+0	[M+Na]+								
PI[36:1]	-ve	863.564	M+0	[M-H]-	2.97 [0-8.56]	n.s.	2.47 [0-8.56]	3.90 [0-7.72]	1.92 [0-15.15]	p<0.001	1.11 [0-1.92]	3.52 [0-15.15]
	+ve	865.581	M+0	[M+H]+								
	+ve	882.605	M+0	[M+NH4]+								
	+ve	883.607	M+1	[M+NH4]+								
	+ve	887.562	M+0	[M+Na]+								
**Glycerolipids**												
DG[34:1]	+ve	612.575	M+0	[M+NH4]+	0.08 [0-0.45]	p=0.037	0.09 [0-0.45]	0.06 [0-0.13]	0.04 [0-0.51]	n.s.	0.03 [0-0.09]	0.05 [0-0.51]
TG[50:2]	+ve	848.764	M+0	[M+NH4]+	0.08 [0-0.83]	n.s.	0.08 [0-0.82]	0.03 [0-0.83]	0.06 [0-0.70]	n.s.	0.07 [0-0.70]	0.04 [0-0.17]
TG[50:1]	+ve	850.778	M+0	[M+NH4]+	0.14 [0-1.32]	n.s.	0.14 [0-0.84]	0.09 [0-1.32]	0.06 [0-0.72]	n.s.	0.06 [0-0.72]	0.07 [0-0.35]
	+ve	851.782	M+1	[M+NH4]+								
	+ve	855.727	M+0	[M+Na]+								
	+ve	856.724*	M+1	[M+Na]+								
TG[50:0]	+ve	852.787	M+0	[M+NH4]+	0.06 [0-1.15]	n.s.	0.07 [0-1.15]	0.06 [0-0.40]	0.03 [0-0.21]	n.s.	0.03 [0-0.21]	0.06 [0-0.09]
TG[52:4]	+ve	872.756	M+0	[M+NH4]+	0.10 [0-0.56]	n.s.	0.12 [0-0.27]	0.07 [0-0.56]	0.04 [0-0.31]	n.s.	0.04 [0-0.28]	0.05 [0-0.31]
	+ve	873.759	M+1	[M+NH4]+								
TG[52:3]	+ve	874.778	M+0	[M+NH4]+	0.13 [0-0.72]	n.s.	0.20 [0-0.72]	0.10 [0-0.58]	0.08 [0-0.62]	n.s.	0.07 [0-0.62]	0.08 [0-0.18]
	+ve	875.779	M+1	[M+NH4]+								
TG[52:2]	+ve	876.795	M+0	[M+NH4]+	0.14 [0-1.37]	n.s.	0.15 [0-0.99]	0.13 [0-1.37]	0.07 [0-0.85]	n.s.	0.06 [0-0.85]	0.12 [0-0.35]
	+ve	877.799	M+1	[M+NH4]+								
	+ve	881.747	M+0	[M+Na]+								
TG[52:1]	+ve	878.806	M+0	[M+NH4]+	0.08 [0-0.71]	n.s.	0.10 [0-0.45]	0.04 [0-0.71]	0.04 [0-0.39]	n.s.	0.03 [0-0.39]	0.04 [0-0.19]
TG[54:5]	+ve	898.768	M+0	[M+NH4]+	0.10 [0-1.16]	n.s.	0.09 [0-0.26]	0.11 [0-1.16]	0.04 [0-1.14]	n.s.	0.04 [0-1.14]	0.06 [0-0.18]
TG[54:4]	+ve	900.792	M+0	[M+NH4]+	0.07 [0-1.55]	n.s.	0.07 [0-0.32]	0.06 [0-1.55]	0.04 [0-1.52]	n.s.	0.04 [0-1.52]	0.05 [0-0.19]
	+ve	901.797	M+1	[M+NH4]+								
TG[54:3]	+ve	902.812	M+0	[M+NH4]+	0.13 [0-1.91]	n.s.	0.13 [0-0.49]	0.07 [0-1.91]	0.07 [0-1.88]	n.s.	0.07 [0-1.88]	0.06 [0-0.22]
	+ve	903.812	M+1	[M+NH4]+								
TG[54:2]	+ve	904.824	M+0	[M+NH4]+	0.07 [0-0.45]	n.s.	0.09 [0-0.19]	0.07 [0-0.45]	0.03 [0-0.44]	n.s.	0.03 [0-0.44]	0.05 [0-0.15]
**Sterol lipids**												
Cholesterol	+ve	369.352	M+0	[M-OH]+	1.89 [0.52-14.52]	p<0.001	2.88 [1.23-14.52]	1.08 [0.52-3.88]	0.95 [0.09-6.26]	p=0.035	0.91 [0.09-5.14]	1.63 [0.45-6.26]
	+ve	370.355	M+1	[M-OH]+								
	+ve	404.383	M+0	[M+NH4]+								
a-Tocopherol	+ve	431.387	M+0	[M+H]+	0.04 [0-1.23]	p<0.001	0.08 [0-1.23]	0.02 [0-0.04]	0.02 [0-0.10]	n.s.	0.02 [0-0.10]	0.03 [0-0.07]
CE[16:1]	+ve	645.551	M+0	[M+NH4]+	0.14 [0-1.18]	p=0.042	0.18 [0-1.18]	0.07 [0-0.16]	0.08 [0-0.64]	n.s.	0.07 [0-0.11]	0.17 [0-0.64]
CE[18:2]	+ve	666.619	M+0	[M+NH4]+	0.22 [0-1.89]	p<0.001	0.49 [0-1.89]	0.11 [0-0.72]	0.11 [0-0.67]	n.s.	0.11 [0-0.65]	0.13 [0-0.67]
	+ve	667.621	M+1	[M+NH4]+								
	+ve	671.575	M+0	[M+Na]+								
	+ve	672.575	M+1	[M+Na]+								
	+ve	687.546	M+0	[M+K]+								
	+ve	688.543	M+1	[M+K]+								

Continued

Lipid					Relative abundance (% of DPPC)				Abundance (µM normalised to DMPC internal standard)			
Assignment	ESI	m/z	Mass isotopomer	Ion	All samples median [range]	MW U test p-value	Group A median [range]	Group B median [range]	All samples median [range]	MW U test p-value	Group A median [range]	Group B median [range]
CE[18:1]	+ve	668.627	M+0	[M+NH4]+	0.13 [0-4.50]	p=0.005	0.20 [0-4.50]	0.09 [0-0.32]	0.06 [0-0.61]	n.s.	0.06 [0-0.46]	0.07 [0-0.61]
	+ve	669.628	M+1	[M+NH4]+								
	+ve	673.588	M+0	[M+Na]+								
	+ve	689.556	M+0	[M+K]+								
**Sphingolipids**												
Putative Cer	+ve	520.507	NA	NA	0.17 [0-0.91]	p<0.001	0.31 [0-0.91]	0.09 [0.02-0.20]	0.09 [0-0.78]	n.s.	0.09 [0-0.43]	0.11 [0.04-0.78]
	+ve	521.512	NA	NA								
Cer[d18:1/16:0]	+ve	538.524	M+0	[M+H]+	0.07 [0-0.94]	p<0.001	0.14 [0-0.94]	0.04 [0-0.09]	0.04 [0-0.43]	n.s.	0.04 [0-0.43]	0.05 [0-0.39]
	+ve	539.529	M+1	[M+H]+								
	+ve	560.504	M+0	[M+Na]+								
Cer[d18:0/16:0]	+ve	540.535	M+0	[M+H]+	0.06 [0-0.09]	p=0.010	0.07 [0-0.09]	0.03 [0-0.07]	0.02 [0-0.10]	n.s.	0.02 [0-0.04]	0.03 [0-0.10]
Cer[d18:1/18:1]	+ve	564.357	M+0	[M+H]+	0.11 [0-1.66]	n.s.	0.14 [0-1.66]	0.10 [0-0.50]	0.04 [0-2.00]	p=0.004	0.03 [0-0.41]	0.16 [0-2.00]
Cer[d18:1/18:0]	+ve	566.554	M+0	[M+H]+	0.06 [0-1.85]	p=0.011	0.09 [0-1.85]	0.04 [0-0.09]	0.03 [0-0.85]	n.s.	0.03 [0-0.85]	0.06 [0-0.38]
	+ve	588.536	M+0	[M+Na]+								
HexCer[d18:1/16:0]	+ve	717.553*	M+0	[M+NH4]+	0.07 [0-0.36]	p=0.017	0.09 [0-0.36]	0.06 [0.03-0.22]	0.04 [0-0.58]	p<0.001	0.03 [0-0.11]	0.09 [0.02-0.58]
	+ve	718.558*	M+1	[M+NH4]+								
Putative HexCer	+ve	728.526	M+0	[M+H]+	0.86 [0.48-1.76]	n.s.	0.80 [0.48-1.76]	0.94 [0.61-1.36]	0.42 [0.04-4.21]	p<0.001	0.29 [0.04-0.60]	1.56 [0.42-4.21]
SM[d18:1/14:0]	+ve	675.544	M+0	[M+H]+	0.06 [0-0.42]	n.s.	0.08 [0-0.42]	0.04 [0-0.26]	0.04 [0-0.56]	n.s.	0.03 [0-0.13]	0.07 [0-0.56]
SM[d18:1/16:0]	+ve	703.572	M+0	[M+H]+	0.76 [0.27-6.37]	p=0.002	1.20 [0.38-6.37]	0.56 [0.27-1.19]	0.40 [0.11-3.74]	p<0.001	0.33 [0.11-0.90]	0.69 [0.25-3.74]
	+ve	704.564*	M+1	[M+H]+								
	+ve	705.578*	M+2	[M+H]+								
SM[d18:1/18:0]	+ve	731.603*	M+0	[M+H]+	0.27 [0-1.28]	n.s.	0.34 [0-1.28]	0.22 [0-0.27]	0.07 [0-0.96]	p=0.018	0.05 [0-0.19]	0.25 [0-0.96]
	+ve	732.554*	M+1	[M+H]+								
Putative SM	+ve	801.678	M+0	NA	0.07 [0-0.41]	p=0.005	0.09 [0-0.41]	0.05 [0-0.14]	0.03 [0-0.45]	p=0.006	0.02 [0-0.09]	0.05 [0-0.45]
	+ve	802.676	M+1	NA								
SM[d18:1/24:1]	+ve	813.681	M+0	[M+H]+	0.25 [0.08-2.36]	p=0.005	0.36 [0.08-2.36]	0.19 [0.09-0.48]	0.12 [0.03-1.14]	p<0.001	0.10 [0.03-0.29]	0.23 [0.10-1.14]
	+ve	814.680	M+1	[M+H]+								
SM[d18:1/24:0]	+ve	815.694	M+0	[M+H]+	0.17 [0-1.47]	p=0.002	0.22 [0-1.47]	0.13 [0.06-0.31]	0.09 [0-0.76]	p<0.001	0.06 [0-0.15]	0.15 [0.06-0.76]
	+ve	816.702	M+1	[M+H]+								
	+ve	817.688	M+2	[M+H]+								
**Unidentified ions and contaminants**												
Unknown	+ve	374.308	NA	NA	0.09 [0-1.63]	p=0.003	0.15 [0-1.63]	0.04 [0-0.19]	0.04 [0-0.15]	n.s.	0.03 [0-0.15]	0.06 [0-0.13]
Unknown	+ve	386.761	NA	NA	0.05 [0-0.11]	p<0.001	0.06 [0-0.11]	0.04 [0-0.06]	0.04 [0-0.14]	p<0.001	0.02 [0-0.06]	0.08 [0-0.14]
Unknown	+ve	388.343	NA	NA	0.54 [0-5.73]	n.s.	0.54 [0-5.73]	0.44 [0-1.00]	0.19 [0-0.79]	n.s.	0.18 [0-0.79]	0.42 [0-0.55]
Unknown	+ve	389.773	NA	NA	0.05 [0-0.22]	n.s.	0.06 [0-0.22]	0.05 [0-0.09]	0.03 [0-0.13]	p=0.005	0.02 [0-0.04]	0.04 [0-0.13]
Unknown	+ve	423.248	NA	NA	0.14 [0-1.05]	p<0.001	0.20 [0.05-1.05]	0.06 [0-0.33]	0.06 [0-0.50]	n.s.	0.06 [0.02-0.21]	0.11 [0-0.50]
Unknown	+ve	440.270	NA	NA	0.14 [0-1.43]	p<0.001	0.19 [0.07-1.43]	0.06 [0-0.43]	0.08 [0-0.33]	n.s.	0.08 [0.02-0.29]	0.11 [0-0.33]
Unknown	+ve	445.224	NA	NA	0.15 [0-1.39]	p=0.002	0.32 [0-1.39]	0.06 [0-0.68]	0.07 [0-0.30]	n.s.	0.08 [0-0.30]	0.06 [0-0.21]
Unknown	+ve	467.206	NA	NA	0.10 [0-1.11]	p=0.023	0.22 [0-1.11]	0.05 [0-0.62]	0.05 [0-0.28]	n.s.	0.04 [0-0.28]	0.05 [0-0.21]
Unknown	+ve	549.508	NA	NA	0.08 [0-2.04]	n.s.	0.14 [0-2.04]	0.06 [0-0.43]	0.05 [0-0.40]	n.s.	0.04 [0-0.18]	0.11 [0-0.40]
Unknown	+ve	551.506	NA	NA	0.18 [0-1.02]	p=0.030	0.23 [0-1.02]	0.16 [0.03-0.31]	0.11 [0-0.50]	p<0.001	0.05 [0-0.17]	0.21 [0.03-0.50]
Unknown	+ve	575.508	NA	NA	0.08 [0-1.10]	n.s.	0.10 [0-1.10]	0.08 [0.03-0.16]	0.06 [0-0.41]	p<0.001	0.03 [0-0.24]	0.14 [0.03-0.41]
Unknown	+ve	577.519	NA	NA	0.71 [0-1.35]	n.s.	0.68 [0-1.35]	0.72 [0.20-1.24]	0.44 [0-2.87]	p<0.001	0.21 [0-0.62]	1.25 [0.44-2.87]
Unknown	+ve	578.524	NA	NA	0.27 [0-0.54]	n.s.	0.25 [0-0.54]	0.28 [0.07-0.45]	0.18 [0-1.14]	p<0.001	0.10 [0-0.23]	0.53 [0.16-1.14]
Unknown	+ve	579.534	NA	NA	0.10 [0-0.29]	n.s.	0.11 [0-0.29]	0.10 [0.07-0.18]	0.07 [0-0.60]	p<0.001	0.03 [0-0.13]	0.21 [0.06-0.60]
Unknown	+ve	601.521	NA	NA	0.07 [0-0.55]	n.s.	0.07 [0-0.55]	0.07 [0-0.35]	0.05 [0-0.47]	p=0.028	0.03 [0-0.17]	0.07 [0.02-0.47]
Unknown	+ve	603.535	NA	NA	0.30 [0-1.20]	n.s.	0.26 [0-1.20]	0.31 [0.12-0.61]	0.18 [0-1.39]	p<0.001	0.08 [0-0.26]	0.52 [0.19-1.39]
Unknown	+ve	604.542	NA	NA	0.13 [0-0.48]	n.s.	0.11 [0-0.48]	0.15 [0-0.25]	0.09 [0-0.74]	p<0.001	0.04 [0-0.13]	0.25 [0.08-0.74]
Unknown	+ve	605.550	NA	NA	0.43 [0-0.85]	n.s.	0.40 [0-0.85]	0.44 [0.23-0.79]	0.23 [0-2.04]	p<0.001	0.13 [0-0.27]	0.73 [0.26-2.04]

Continued

Lipid					Relative abundance (% of DPPC)				Abundance (µM normalised to DMPC internal standard)			
Assignment	ESI	m/z	Mass isotopomer	Ion	All samples median [range]	MW U test p-value	Group A median [range]	Group B median [range]	All samples median [range]	MW U test p-value	Group A median [range]	Group B median [range]
Unknown	+ve	606.551	NA	NA	0.17 [0-0.31]	n.s.	0.17 [0-0.31]	0.17 [0.08-0.30]	0.09 [0-0.96]	p<0.001	0.05 [0-0.10]	0.31 [0.09-0.96]
Unknown	+ve	627.556	NA	NA	0.05 [0-0.70]	n.s.	0.08 [0-0.70]	0.04 [0.01-0.20]	0.04 [0-0.57]	n.s.	0.04 [0-0.10]	0.05 [0.01-0.57]
Unknown	+ve	640.591	NA	NA	0.08 [0-1.86]	p=0.005	0.12 [0-1.86]	0.06 [0-0.14]	0.04 [0-0.56]	n.s.	0.04 [0-0.16]	0.05 [0-0.56]
Unknown	+ve	641.592	NA	NA	0.09 [0-1.26]	n.s.	0.12 [0-1.26]	0.06 [0-0.21]	0.04 [0-0.55]	n.s.	0.04 [0-0.10]	0.11 [0-0.55]
Unknown	+ve	642.604	NA	NA	0.09 [0-0.57]	n.s.	0.09 [0-0.57]	0.06 [0-0.32]	0.03 [0-0.56]	n.s.	0.03 [0-0.16]	0.04 [0-0.56]
Unknown	+ve	648.659	NA	NA	0.06 [0-0.18]	n.s.	0.07 [0-0.18]	0.06 [0-0.14]	0.02 [0-0.56]	p=0.001	0.02 [0-0.06]	0.14 [0-0.56]
Unknown	+ve	662.569	NA	NA	0.06 [0-0.19]	n.s.	0.07 [0-0.19]	0.03 [0-0.14]	0.02 [0-0.58]	p=0.033	0.02 [0-0.06]	0.11 [0-0.58]
Unknown	+ve	664.596	NA	NA	0.06 [0-0.31]	n.s.	0.07 [0-0.31]	0.05 [0-0.12]	0.03 [0-0.55]	p=0.001	0.02 [0-0.12]	0.09 [0-0.55]
Unknown	+ve	671.445	NA	NA	0.18 [0-4.71]	p=0.002	0.33 [0-4.71]	0.09 [0-0.39]	0.08 [0-0.90]	p=0.025	0.07 [0-0.18]	0.16 [0-0.90]
Unknown	+ve	672.447	NA	NA	0.14 [0-2.23]	p=0.020	0.16 [0-2.23]	0.08 [0-0.25]	0.05 [0-1.01]	p=0.006	0.03 [0-0.08]	0.08 [0-1.01]
Unknown	+ve	695.575	NA	NA	0.10 [0-0.76]	n.s.	0.13 [0-0.76]	0.06 [0-0.26]	0.05 [0-0.28]	n.s.	0.04 [0-0.28]	0.09 [0-0.20]
Unknown	+ve	723.536	NA	NA	0.10 [0-0.23]	n.s.	0.10 [0-0.23]	0.10 [0.06-0.16]	0.07 [0-0.66]	p<0.001	0.04 [0-0.09]	0.19 [0.05-0.66]
Unknown	+ve	724.524	NA	NA	0.21 [0.09-1.08]	p=0.004	0.27 [0.12-1.08]	0.16 [0.09-0.31]	0.11 [0.01-1.08]	p<0.001	0.08 [0.01-0.23]	0.23 [0.08-1.08]
Unknown	+ve	725.539	NA	NA	0.13 [0.05-0.74]	p=0.007	0.18 [0.07-0.74]	0.11 [0.05-0.21]	0.07 [0.02-0.89]	p<0.001	0.06 [0.02-0.16]	0.14 [0.04-0.89]
Unknown	+ve	726.544	NA	NA	0.12 [0-0.91]	p<0.001	0.17 [0-0.91]	0.10 [0.04-0.18]	0.06 [0-0.76]	p<0.001	0.05 [0-0.10]	0.12 [0.05-0.76]
Unknown	+ve	727.528	NA	NA	0.08 [0-0.39]	p=0.002	0.10 [0-0.39]	0.03 [0-0.11]	0.03 [0-0.50]	p=0.006	0.03 [0-0.04]	0.04 [0-0.50]
Unknown	+ve	729.533	NA	NA	0.40 [0.23-1.27]	n.s.	0.40 [0.23-1.27]	0.40 [0.26-0.60]	0.20 [0.03-1.84]	p<0.001	0.14 [0.03-0.29]	0.69 [0.19-1.84]
Unknown	+ve	752.557	NA	NA	0.29 [0.13-1.68]	p=0.001	0.38 [0.19-1.68]	0.23 [0.13-0.43]	0.15 [0.03-1.35]	p<0.001	0.12 [0.03-0.29]	0.38 [0.12-1.35]
Unknown	+ve	753.561	NA	NA	0.10 [0-0.51]	n.s.	0.11 [0-0.51]	0.10 [0.05-0.21]	0.06 [0-0.84]	p<0.001	0.05 [0-0.12]	0.18 [0.04-0.84]
Unknown	+ve	770.575	NA	NA	0.34 [0.23-1.19]	n.s.	0.34 [0.23-1.19]	0.35 [0.24-0.50]	0.18 [0.03-1.67]	p<0.001	0.11 [0.03-0.24]	0.64 [0.16-1.67]
Unknown	+ve	779.591	NA	NA	0.09 [0-0.96]	n.s.	0.17 [0-0.96]	0.07 [0.03-0.16]	0.06 [0-0.63]	p<0.001	0.03 [0-0.09]	0.14 [0.03-0.63]
Unknown	+ve	799.557	NA	NA	0.13 [0-0.53]	n.s.	0.17 [0-0.53]	0.13 [0-0.29]	0.11 [0-0.63]	p=0.002	0.05 [0-0.12]	0.18 [0.04-0.63]
Unknown	+ve	800.562	NA	NA	0.08 [0-0.18]	n.s.	0.07 [0-0.17]	0.08 [0.03-0.18]	0.06 [0-0.50]	p<0.001	0.03 [0-0.06]	0.11 [0.03-0.50]
Unknown	+ve	800.659	NA	NA	0.07 [0-0.18]	n.s.	0.09 [0-0.18]	0.07 [0-0.11]	0.03 [0-0.44]	n.s.	0.03 [0-0.06]	0.14 [0-0.44]
Unknown	+ve	802.564	NA	NA	0.06 [0-0.15]	n.s.	0.07 [0-0.15]	0.06 [0.03-0.11]	0.04 [0-0.30]	p<0.001	0.03 [0-0.06]	0.09 [0-0.30]
Unknown	+ve	803.672	NA	NA	0.11 [0-0.55]	p<0.001	0.15 [0-0.55]	0.07 [0-0.11]	0.04 [0-0.45]	p=0.010	0.02 [0-0.05]	0.09 [0-0.45]
Unknown	+ve	821.529	NA	NA	0.08 [0-0.16]	n.s.	0.10 [0-0.15]	0.07 [0.03-0.16]	0.05 [0-0.47]	p<0.001	0.03 [0-0.07]	0.10 [0.03-0.47]
Unknown	+ve	822.527	NA	NA	0.12 [0-0.24]	n.s.	0.12 [0-0.24]	0.12 [0.09-0.18]	0.07 [0-0.64]	p<0.001	0.04 [0-0.11]	0.22 [0.07-0.64]
Unknown	+ve	823.533	NA	NA	0.08 [0-0.15]	n.s.	0.08 [0-0.15]	0.08 [0.05-0.12]	0.04 [0-0.47]	p<0.001	0.03 [0-0.07]	0.14 [0.04-0.47]
Unknown	+ve	824.539	NA	NA	0.57 [0.34-1.18]	n.s.	0.57 [0.34-1.18]	0.58 [0.41-0.80]	0.29 [0.03-2.46]	p<0.001	0.16 [0.03-0.50]	1.02 [0.27-2.46]
Unknown	+ve	825.543	NA	NA	0.27 [0.16-0.67]	n.s.	0.27 [0.16-0.67]	0.27 [0.19-0.37]	0.14 [0.02-1.24]	p<0.001	0.08 [0.02-0.23]	0.49 [0.14-1.24]
Unknown	+ve	826.548	NA	NA	0.10 [0-0.18]	n.s.	0.11 [0-0.18]	0.09 [0.07-0.15]	0.06 [0-0.56]	p<0.001	0.05 [0-0.10]	0.18 [0.05-0.56]
Unknown	+ve	830.570	NA	NA	0.07 [0-0.12]	n.s.	0.08 [0-0.12]	0.07 [0-0.11]	0.04 [0-0.37]	p<0.001	0.03 [0-0.05]	0.08 [0-0.37]
Unknown	+ve	836.548	NA	NA	0.08 [0-0.41]	n.s.	0.10 [0-0.41]	0.07 [0-0.16]	0.05 [0-0.60]	p=0.020	0.04 [0-0.12]	0.11 [0-0.60]
Unknown	+ve	837.549	NA	NA	0.12 [0-0.31]	p=0.030	0.16 [0-0.31]	0.11 [0.05-0.23]	0.06 [0-0.61]	p<0.001	0.05 [0-0.08]	0.18 [0.04-0.61]
Unknown	+ve	838.554	NA	NA	0.10 [0-0.30]	n.s.	0.11 [0-0.30]	0.09 [0.05-0.19]	0.06 [0-0.55]	p<0.001	0.05 [0-0.08]	0.19 [0.04-0.55]
Unknown	+ve	844.617	NA	NA	0.09 [0-0.45]	p<0.001	0.18 [0-0.45]	0.06 [0-0.10]	0.05 [0-0.40]	p=0.001	0.03 [0-0.05]	0.08 [0-0.40]
Unknown	+ve	848.539	NA	NA	0.07 [0-0.13]	n.s.	0.07 [0-0.13]	0.07 [0.04-0.11]	0.05 [0-0.41]	p<0.001	0.03 [0-0.06]	0.12 [0.04-0.41]
Unknown	+ve	850.554	NA	NA	0.12 [0-0.23]	n.s.	0.12 [0-0.23]	0.12 [0.10-0.17]	0.07 [0-0.62]	p<0.001	0.05 [0-0.12]	0.23 [0.07-0.62]
Unknown	+ve	851.559	NA	NA	0.08 [0-0.36]	n.s.	0.08 [0-0.36]	0.08 [0.06-0.11]	0.04 [0-0.42]	p<0.001	0.03 [0-0.10]	0.14 [0.04-0.42]
Unknown	+ve	852.566	NA	NA	0.07 [0-0.26]	n.s.	0.08 [0-0.26]	0.06 [0.05-0.10]	0.04 [0-0.38]	p<0.001	0.02 [0-0.05]	0.11 [0.04-0.38]
Unknown	+ve	854.576	NA	NA	0.06 [0-0.15]	n.s.	0.06 [0-0.11]	0.06 [0-0.15]	0.04 [0-0.34]	p<0.001	0.03 [0-0.05]	0.10 [0-0.34]
Unknown	+ve	858.527	NA	NA	0.06 [0-0.13]	n.s.	0.07 [0-0.13]	0.06 [0-0.10]	0.03 [0-0.42]	p=0.002	0.03 [0-0.05]	0.09 [0-0.42]
Unknown	+ve	858.715	NA	NA	0.06 [0-0.13]	n.s.	0.06 [0-0.13]	0.05 [0-0.08]	0.03 [0-0.22]	p=0.001	0.02 [0-0.05]	0.04 [0-0.22]
Unknown	+ve	861.544	NA	NA	0.06 [0-0.11]	p=0.014	0.07 [0-0.11]	0.05 [0-0.09]	0.04 [0-0.34]	p=0.009	0.02 [0-0.04]	0.08 [0-0.34]
Unknown	+ve	862.626	NA	NA	0.07 [0-0.74]	p=0.042	0.09 [0-0.74]	0.04 [0-0.11]	0.03 [0-0.39]	n.s.	0.03 [0-0.07]	0.07 [0-0.39]
Unknown	+ve	866.585	NA	NA	0.06 [0-0.17]	n.s.	0.06 [0-0.14]	0.07 [0.03-0.17]	0.04 [0-0.41]	p<0.001	0.02 [0-0.05]	0.10 [0.03-0.41]
Unknown	+ve	868.687	NA	NA	0.09 [0-0.57]	n.s.	0.09 [0-0.57]	0.06 [0-0.21]	0.03 [0-0.28]	p=0.002	0.02 [0-0.07]	0.08 [0-0.28]
Unknown	+ve	869.694	NA	NA	0.06 [0-0.14]	n.s.	0.07 [0-0.14]	0.05 [0-0.11]	0.03 [0-0.20]	p=0.022	0.02 [0-0.06]	0.05 [0-0.20]
Unknown	+ve	876.562	NA	NA	0.06 [0-0.11]	n.s.	0.06 [0-0.11]	0.05 [0.03-0.10]	0.04 [0-0.35]	p<0.001	0.02 [0-0.08]	0.09 [0.02-0.35]

Continued

Lipid					Relative abundance (% of DPPC)				Abundance (µM normalised to DMPC internal standard)			
Assignment	ESI	m/z	Mass isotopomer	Ion	All samples median [range]	MW U test p-value	Group A median [range]	Group B median [range]	All samples median [range]	MW U test p-value	Group A median [range]	Group B median [range]
Unknown	+ve	884.607	NA	NA	0.03 [0-0.08]	n.s.	0.05 [0-0.08]	0.03 [0-0.07]	0.02 [0-0.22]	p=0.005	0.02 [0-0.03]	0.05 [0-0.22]
Unknown	+ve	886.560	NA	NA	0.11 [0-0.63]	p<0.001	0.15 [0-0.63]	0.08 [0.04-0.16]	0.07 [0-0.34]	p<0.001	0.04 [0-0.22]	0.12 [0.05-0.34]
Unknown	+ve	888.567	NA	NA	0.05 [0-0.11]	n.s.	0.05 [0-0.10]	0.05 [0-0.11]	0.04 [0-0.28]	p=0.002	0.03 [0-0.05]	0.06 [0.02-0.28]
Unknown	+ve	889.587	NA	NA	0.05 [0-0.67]	n.s.	0.08 [0-0.67]	0.03 [0-0.12]	0.03 [0-0.24]	p=0.030	0.02 [0-0.10]	0.05 [0-0.24]
Unknown	+ve	892.530	NA	NA	0.06 [0-0.12]	p=0.041	0.06 [0-0.12]	0.05 [0.03-0.09]	0.04 [0-0.35]	p<0.001	0.03 [0-0.07]	0.09 [0.02-0.35]
Unknown	+ve	900.578	NA	NA	0.05 [0-0.11]	p=0.002	0.07 [0-0.11]	0.05 [0-0.07]	0.03 [0-0.28]	p<0.001	0.02 [0-0.05]	0.06 [0-0.28]
Unknown	+ve	902.570	NA	NA	0.06 [0-0.61]	p=0.011	0.08 [0-0.61]	0.05 [0.02-0.08]	0.04 [0-0.27]	p<0.001	0.02 [0-0.06]	0.08 [0.02-0.27]
Unknown	+ve	904.586	NA	NA	0.06 [0-0.38]	p=0.003	0.09 [0-0.38]	0.05 [0.03-0.16]	0.04 [0-0.27]	p<0.001	0.03 [0-0.06]	0.09 [0.03-0.27]
Unknown	+ve	905.591	NA	NA	0.05 [0-0.24]	n.s.	0.07 [0-0.24]	0.04 [0-0.10]	0.02 [0-0.24]	p=0.003	0.02 [0-0.04]	0.05 [0-0.24]
Unknown	+ve	909.546	NA	NA	0.05 [0-0.13]	n.s.	0.07 [0-0.09]	0.04 [0-0.13]	0.04 [0-0.27]	p=0.003	0.02 [0-0.05]	0.07 [0-0.27]
Unknown	+ve	912.581	NA	NA	0.08 [0-0.28]	p=0.002	0.12 [0-0.28]	0.06 [0-0.13]	0.05 [0-0.24]	p=0.001	0.03 [0-0.17]	0.08 [0-0.24]
Unknown	+ve	914.591	NA	NA	0.08 [0-0.39]	p<0.001	0.14 [0-0.39]	0.07 [0-0.19]	0.07 [0-0.27]	p=0.001	0.04 [0-0.27]	0.09 [0-0.25]
Unknown	+ve	930.602	NA	NA	0.06 [0-0.23]	p=0.001	0.07 [0-0.23]	0.04 [0-0.07]	0.03 [0-0.21]	p<0.001	0.02 [0-0.06]	0.06 [0-0.21]
Unknown	+ve	972.720	NA	NA	0.08 [0-0.83]	p=0.017	0.11 [0-0.83]	0.07 [0-0.10]	0.04 [0-0.29]	p=0.001	0.03 [0-0.05]	0.10 [0-0.29]
Unknown	+ve	973.723	NA	NA	0.06 [0-0.45]	p=0.008	0.09 [0-0.45]	0.05 [0-0.07]	0.03 [0-0.18]	p=0.004	0.02 [0-0.03]	0.06 [0-0.18]
Unknown	+ve	1024.674	NA	NA	0.06 [0-0.11]	NA	0.06 [0-0.11]	0.04 [0-0.04]	0.02 [0-0.03]	NA	0.02 [0-0.03]	not detected
Unknown	-ve	421.226	NA	NA	26.44 [0-431.86]	n.s.	26.32 [0-278.73]	26.44 [0-431.86]	14.54 [0-111.20]	n.s.	15.88 [0-111.20]	7.95 [0-63.64]
Unknown	-ve	422.229	NA	NA	6.55 [0-93.83]	n.s.	15.31 [0-59.98]	6.33 [0-93.83]	3.68 [0-24.16]	n.s.	3.68 [0-24.16]	3.39 [0-14.26]
Unknown	-ve	489.244	NA	NA	6.70 [0-66.00]	n.s.	7.23 [0-66.00]	3.89 [0-39.37]	3.68 [0-12.46]	n.s.	3.63 [0-9.10]	4.11 [0-12.46]
Unknown	-ve	517.283	NA	NA	4.36 [0-44.23]	n.s.	5.28 [0-44.23]	2.12 [0-25.31]	2.06 [0-8.01]	n.s.	1.85 [0-6.00]	2.66 [0-8.01]
Unknown	-ve	582.510	NA	NA	1.97 [0-16.76]	p=0.018	4.02 [0-16.76]	1.19 [0-5.88]	1.11 [0-10.58]	n.s.	0.84 [0-5.25]	2.21 [0-10.58]
Unknown	-ve	768.532	NA	NA	2.31 [0-29.74]	n.s.	1.89 [0-29.74]	2.61 [0-22.27]	2.33 [0-19.74]	p=0.007	1.15 [0-5.38]	4.10 [0-19.74]
Unknown	-ve	777.550	NA	NA	7.14 [0-9.35]	n.s.	7.03 [0-9.35]	8.03 [0-9.26]	2.93 [0-20.15]	p<0.001	2.20 [0-5.41]	7.99 [0-20.15]
Unknown	-ve	836.529	NA	NA	1.61 [0-9.47]	n.s.	1.54 [0-9.21]	1.99 [0-9.47]	1.81 [0-8.66]	p=0.006	0.81 [0-2.18]	2.59 [0-8.66]
Unknown	-ve	846.548	NA	NA	12.19 [0-17.86]	n.s.	12.29 [0-17.86]	12.06 [0-17.08]	4.78 [0-24.28]	p<0.001	3.02 [0-6.50]	10.30 [0-24.28]
Unknown	-ve	847.551	NA	NA	4.92 [0-7.77]	n.s.	4.78 [0-7.34]	5.69 [0-7.77]	2.47 [0-11.46]	p<0.001	1.61 [0-2.98]	4.64 [0-11.46]
Plasticiser	+ve	637.306	NA	NA	0.25 [0-3.07]	p<0.001	0.43 [0-3.07]	0.15 [0-0.27]	0.11 [0-0.28]	n.s.	0.11 [0.05-0.28]	0.14 [0-0.22]
	+ve	638.308	NA	NA								
Plasticiser	+ve	654.334	NA	NA	0.28 [0.04-3.68]	p<0.001	0.53 [0.10-3.68]	0.20 [0.04-0.35]	0.15 [0.06-1.19]	p=0.008	0.13 [0.06-0.36]	0.23 [0.07-1.19]
	+ve	655.339	NA	NA								
Plasticiser	+ve	659.287	NA	NA	0.09 [0-1.36]	p=0.001	0.12 [0-1.36]	0.05 [0-0.11]	0.03 [0-0.10]	n.s.	0.03 [0-0.05]	0.04 [0-0.10]
Plasticiser	+ve	771.528	NA	NA	0.31 [0-1.32]	n.s.	0.35 [0-1.32]	0.22 [0-0.63]	0.17 [0-0.97]	p<0.001	0.13 [0-0.18]	0.38 [0.16-0.97]
